# Analysis of differences in intestinal flora associated with different BMI status in colorectal cancer patients

**DOI:** 10.1186/s12967-024-04903-7

**Published:** 2024-02-09

**Authors:** Yongqi Huang, Xiaoliang Huang, Zhen Wang, Fuhai He, Zigui Huang, Chuanbin Chen, Binzhe Tang, Mingjian Qin, Yongzhi Wu, Chenyan Long, Weizhong Tang, Xianwei Mo, Jungang Liu

**Affiliations:** Division of Colorectal and Anal Surgery, Department of Gastrointestinal Surgery, Guangxi Medical University Cancer Hospital, Nanning, People’s Republic of China

**Keywords:** Colorectal cancer, BMI status, 16S rRNA, Intestinal flora

## Abstract

**Background:**

Overweight is known to be an important risk factor for colorectal cancer (CRC), and the differences in intestinal flora among CRC patients with different BMI status have not been clearly defined. The purpose of this study was to elucidate the differences in the abundance, composition and biological function of intestinal flora in CRC patients with different BMI status.

**Method:**

A total of 170 CRC patients were included and grouped according to the BMI data of CRC patients. BMI ≥ 24 kg/m^2^ was defined as overweight group, and BMI within the range of 18.5–23.9 kg/m^2^ was defined as normal weight group. Preoperative stool collection of patients in both groups was used for 16S rRNA sequencing. Total RNA was extracted from 17 CRC tumor tissue samples for transcriptome sequencing, and then CIBERSORT algorithm was used to convert the transcriptome data into the relative content matrix of 22 kinds of immune cells, and the correlation between different intestinal flora and immune cells and immune-related genes under different BMI states was analyzed. Finally, we identified BMI-related differential functional pathways and analyzed the correlation between these pathways and differential intestinal flora.

**Result:**

There was no significant difference in α diversity and β diversity analysis between overweight group and normal weight group. Partial least square discriminant analysis (PLS-DA) could divide the flora into two different clusters according to BMI stratification. A total of 33 BMI-related differential flora were identified by linear discriminant effect size analysis (LEfSe), among which Actinomyces, Desulfovibrio and Bacteroides were significantly enriched in overweight group. ko00514: Other types of O-glycan biosynthesis are significantly enriched in overweight group. There was a significant positive correlation between Clostridium IV and Macrophages M2 and T cells regulatory (Tregs). There was a significant negative correlation with Dendritic cells activated and T cells CD4 memory activated.

**Conclusions:**

The richness and diversity of intestinal flora of CRC patients may be related to different BMI status, and the enrichment of Actinomyces, Desulphurvibrio and Bacteroides may be related to overweight status of CRC patients. The tumor microenvironment in which BMI-related differential flora resides has different immune landscapes, suggesting that some intestinal flora may affect the biological process of CRC by regulating immune cell infiltration and immune gene expression, but further experiments are needed to confirm this.

**Supplementary Information:**

The online version contains supplementary material available at 10.1186/s12967-024-04903-7.

## Introduction

Colorectal cancer (CRC) is a malignant disease that poses a serious threat to human health, and according to the 2020 global cancer statistics released by the International Agency for Cancer (IARC), the incidence of CRC is ranked as the third most prevalent, and about 900,000 people die from CRC each year worldwide, making it the second leading cause of cancer-related deaths [1]. Currently, a significant familial predisposition has been found for some CRC [2], but most sporadic CRC are still mainly attributed to a combination of multiple external environmental factors, such as dietary habits, overweight, and smoking. Overweight is a global health problem that has been shown to be closely associated with the development of various cardiovascular diseases [3]. Nowadays, more and more epidemiological studies have found that there is a correlation between overweight and CRC incidence due to chronic physical inactivity and poor dietary habits [4], and overweight status in adults increases the risk of CRC by 1.2–2 times, and the incidence of CRC associated with overweight accounts for about 14–35% of the total incidence of CRC [5, 6]. There are complex links and mechanisms between overweight and CRC. It has been pointed out that overweight adipose tissue can release higher levels of pro-inflammatory cytokines, such as tumour necrosis factor-alpha (TNF-α) and interleukin-6 (IL-6), which mediate the emergence of CRC-inducing factors, such as chronic inflammation in the organism [7, 8], and that some of the insulin-like adipokines interact with insulin-like growth factor receptor, thereby promoting cancer cells [9]. In addition, overweight-associated insulin resistance, chronic hyperinsulinemia, and intestinal microbial imbalance may also be the underlying pathogenesis of CRC [10–12]. The human gut microbiota consists of 10^13^ to 10^14^ microorganisms, which cover many types of bacteria, fungi, viruses, and other microorganisms, and carries 100 times more genes than the human genome [13]. In the normal human intestinal environment, intestinal microorganisms not only assist in the digestion and absorption of nutrients, but also jointly construct and maintain the intestinal barrier function [14], and play an important role in the regulation of the human immune system to resist the attack of pathogenic bacteria [15, 16]. A growing number of studies have shown that intestinal flora is one of the important factors contributing to CRC [17]. A study that part of the gut microbes release of antigen can activate mitochondrial mediated reactive oxygen molecules, can cause the host cell DNA damage and the activation of oncogene [18]. The intestinal flora of healthy people mainly consists of Firmicutes, Proteobacteria, and Bacteroidetes [19], among which Lactobacillus can provide short-chain fatty acids (SCFAs) such as propionic acid and butyric acid to the intestinal epithelial cells and help to maintain the balance of the intestinal microecology [20, 21], and Bacteroides fragilis, which has been found to be significantly enriched in the intestinal microecology of CRC patients, can activate NF- κB (nuclear factor κB) and induce the development of CRC via TLR4 (Toll- Like Receptor 4 (TLR4) activates NF- κB (nuclear factor κB) and induces inflammation and promotes colon tumour growth [22, 23], and when the intestinal microecosystem is imbalanced, the elevated species and abundance of harmful bacteria may be able to induce CRC through the production of deleterious metabolites and alteration of host physiological processes [24]. Therefore, the species and relative abundance of intestinal microorganisms play an important role in maintaining intestinal microecological stability and human health. With the continuous improvement of living standards, people's living habits and dietary structure have changed dramatically. Excessive intake of high-calorie, high-fat and low-fibre foods, as well as the lack of exercise have led to overweight and intestinal microecological imbalance [25, 26], which increase the risk of developing CRC. Currently, there are few studies on the differences in intestinal flora in CRC patients with different BMI status. Therefore, in order to further explore the relationship between BMI, intestinal flora and CRC, and analyze the differences of intestinal flora in CRC patients under different BMI states, we collected 236 stool samples and 17 tumor samples from CRC patients for 16S rRNA sequencing and transcriptome sequencing, respectively. 16S rRNA sequencing is a method commonly used to identify complex bacterial communities by amplifying the 16S rRNA genes in a sample and then comparing the amplified genes with the known bacterial 16S rRNA gene sequence library to determine which bacteria are present in the sample. Transcriptome sequencing of tumor tissue samples can help to understand the functional status of immune cells and the expression of immune genes in tumor tissues. Combining 16S rRNA sequencing with transcriptome sequencing can help us to correlate intestinal flora and BMI status with tumor immune microenvironment and biological functional pathways, which will help explain the relationship between BMI, intestinal flora and CRC, and explain the biological processes of CRC and intestinal flora under different BMI states.

## Methods

### Subject information and sample collection

The basic information of the subjects and the sample collection protocol were approved by the Medical Ethics Committee of the Affiliated Cancer Hospital of Guangxi Medical University, and all study subjects signed the informed consent form before sample collection. The fecal samples for this study were collected from 236 CRC patients before receiving treatment in the Affiliated Cancer Hospital of Guangxi Medical University between 2021.01.01 and 2021.12.31, and 198 fecal samples were ultimately qualified for 16S rRNA sequencing after screening, of which a total of 170 CRC patients with complete BMI data met the study requirements. Fresh tissue specimens from the above subjects who underwent surgical treatment at the Affiliated Cancer Hospital of Guangxi Medical University were preserved in liquid nitrogen. Subject inclusion criteria: 1. Colorectal cancer patients confirmed by colonoscopic pathological biopsy; 2. No other malignant tumours in combination or in the past; 3. Excluding other intestinal diseases, no acute comorbidities, such as complete intestinal obstruction, intestinal perforation, etc.; 4. All the subjects did not receive any anti-tumour treatment, such as surgical treatment, chemotherapy, radiotherapy, immunotherapy, traditional Chinese medicine, etc., prior to the collection of the stool samples. 5. No antibiotics and other intestinal microecological agents have been used within one month; 6. No impairment of consciousness or other cognitive dysfunction. The CRC patients included in the study were grouped according to their BMI data, with BMI ≥ 24 kg/m^2^ defined as overweight group and BMI within the range of 18.5–23.9 kg/m^2^ defined as normal weight group. Before treatment, patients were instructed to use sterile stool collection tubes to preserve the stool specimens, which were then placed in a sterile ice box, dispensed into 2 ml EP tubes, and finally stored in a − 80 °C refrigerator. For the collection of tissue samples, soybean-sized fresh tissue specimens were isolated from surgically resected tumours and paraneoplastic tissues, and the time from tissue isolation to storage in liquid nitrogen did not exceed 30 min.

### 16S rRNA sequencing

We used the MOBIO PowerSoil^®^ DNA isolation kit to extract sample DNA by adding 200 mg of faecal samples to Tris–EDTA buffer, which was required to check the quality of sample DNA at the end of the DNA extraction. Subsequently, the V3 and V4 regions of the 16S rRNA gene were captured using 341F: (5 ʹ -CCTACGGGGNGGCWGCAG-3 ʹ) and 805R: (5ʹ-GACTACHVGGGGTATCTAATCC-3), and the captured regions were amplified by PCR. The PCR amplification products were detected by 2% agarose gel electrophoresis. The strip size of the captured products was about 300–350 bp and the sequencing depth was 50,000 reads. Then Assay Kit (Catalog Number: P7589) was used to quantify the PCR product. All sample libraries were quantified using KAPA library quantification kit KK 4824. Finally, the library was sequenced using 2 × 250 bp chemistry on the Illumina PE 250 platform.

### Transcriptome sequencing

Total RNA was isolated from 17 CRC tumour tissue specimens using the Trizol^®^ Total RNA Extraction Kit, then RNA integrity was examined using electrophoresis and the purity of the RNA samples was tested using a micro UV spectrophotometer. After removing the rRNA, the cDNA Library was constructed with reference to the instructions for the RNA-seq Sample Preparation Kit (VAHTS™ Stranded mRNA-seq Library Prep Kit for Illumina^®^). The transcriptome libraries were sequenced using the Illumina NovaSeq 6000 platform with 6G of sequencing data per sample, and the quality of the raw sequencing data was evaluated using FastQC. Valid data for all samples were matched with the reference genome (version: hg38) using HISAT2 to assess gene expression. Gene expression was then evaluated using StringTie and known gene models, and finally, per million transcripts (TPM) of each gene was calculated as the expression abundance of the gene.

### Analysis of the CRC tumour immune microenvironment

CIBERSORT is a computational method for analysing the composition of immune cells from RNA sequencing data, which is based on the expression profiles of immune cell-specific genes and uses machine-learning algorithms to analyse and classify the expression patterns of these genes [27]. We use CIBERSORT R script v1.03 to build a model based on support vector regression, utilize the known reference gene expression data and the gene expression data of mixed samples to be estimated, construct an optimization problem through the correlation matrix composed of cells, and solve it in the form of sparse solutions. Thus, the cell composition ratio of the mixed sample is estimated. The TPM matrix obtained by transcriptome sequencing was converted into the relative content matrix of 22 different types and functional states of immune cells. The flora matrix and immune cell abundance matrix were combined, and the correlation coefficients between the columns in the combination matrix were calculated by calling the rcorr function. The type of correlation coefficient was Pearson correlation coefficient.

### Functional enrichment analysis of BMI-related transcriptome sequencing

It is used to assess the enrichment of gene sets in a single sample and to help understand the relevance of gene function, biological processes and disease [28]. We downloaded the required genome files in GMT format (c5.go.v2022.1.Hs.symbols.gmt, c2.cp.kegg.v2022.1.Hs.symbols.gmt). The ssgsea algorithm in the GSVA package v1.46.0 was used to calculate the gene set scoring matrix for each sample. Finally, we used the limma algorithm in the TCGABiolinks package v2.25.3 to analyse the difference KEGG (Kyoto Encyclopedia of Genes and Genomes) pathways with GO (Gene Ontology) entries between the two groups. The GO analysis mainly includes cellular components (CC), molecular functions (MF), and biological process (BP) three main areas. Significance threshold for genes with expression differences: P < 0.05 and |log2FC|> 0.

### 16S rRNA sequencing analysis methods

After obtaining raw sequencing data in FASTQ format for each sample, we quality controlled the raw sequencing data using Quantitative Insights Into Microbial Ecology version 2 (QIIME2), an open source software package for analysing microbial communities. Species annotation of the intestinal flora was performed using the Greengene database v13.8, and then ASV/OUT data of the intestinal flora were extracted using the phyloseq package v1.26.1. We used α-diversity indices to describe the diversity of the intestinal flora communities, where Chao1 and ACE indices were used to compare species abundance and assess relative differences in species diversity between samples, and Shannon and Simpson indices were used to describe species richness, homogeneity, and concentration reflecting species diversity, respectively. β-diversity analyses were used to investigate the species diversity of the intestinal flora between samples. differences in species composition and abundance of intestinal flora between samples, we used the mixOmics package v6.6.2 for partial least squares discriminant analysis (PLS-DA), and the vegan software package v2.5.6 for ADONIS analysis. Linear discriminant analysis effect size (LEfSe) analyses were performed using lefse software v1.0.0 to screen for species most likely to explain differences between groups, while LDA scores were used to assess the effect size of species that differed significantly between groups, with |LDA|> 2 and P < 0.05 as the thresholds for differences to screen for differing species, and the ggplot 2 package v3.4.0 was used to analyse the results as bar charts. The results are presented as bar graphs. We used the PICRUSt 2 analysis tool v2.3.0 to predict KEGG pathway enrichment in overweight and normal weight CRC patients. The α-diversity, β-diversity index and KEGG pathway were compared between the two groups by vegan software package v2.5.6 using Wilcoxon rank sum test. All of the above analyses were performed in R software v3.5.1, all of the above P-values were two-tailed tests, and differences were considered statistically significant when P < 0.05.

### Statistical methods

Statistical analyses were performed using R software v4.2.2 with Pearson chi-square test for categorical variables and t-test for continuous variables in the clinical baseline data. The Hmisc software package v4.7 was loaded and correlations between intestinal flora and immune cell abundance with immune-related genes were calculated using Pearson correlation analysis. The ggcorrplot package v0.1.4 was loaded and the correlation between the dominant microbiota and the enriched BP term, MF term and KEGG pathway was analysed for both groups using Spearman correlation. Finally, the results of the analysis were visualised using pheatmap v1.0.8, ggcorrplot software package v0.1.4, Igraph software package v1.3.5 and Cystoscope software v3.7.2.

## Result

### Basic information and clinical characteristics of CRC patients included in the study

A total of 198 pre-treatment faecal samples from eligible CRC patients were collected for 16S rRNA sequencing in this study, of which 170 CRC patients with complete BMI data contained 108 normal weight CRC patients and 62 overweight CRC patients. As shown in Table 1, there were no statistically significant differences in age and gender between the overweight and normal weight groups of CRC patients, indicating that the baseline data of the CRC patients participating in this study were balanced and comparable. There was no statistical difference between the two groups in terms of TNM stage, perineural infiltration and lymph vascular infiltration (P > 0.05).

**Table 1:** Demographic and clinical characteristics of CRC patients stratified by BMI condition

	Normal weight CRC patients (n = 108)	Overweight CRC patients (n = 62)	P value	Test
Age [year, mean (SD)]	58.66 ± 11.80	57.02 ± 10.84	0.370	T-Test
Age (%)				
< 60	57 (52.8)	38 (61.3)	0.360	Pearson Chi-square
≥ 60	51 (47.2)	24 (38.7)		
Gender (%)				
Male	62 (57.4)	41 (66.1)	0.339	Pearson Chi-square
Female	46 (42.6)	21 (33.9)		
TNM stage (%)				
0	1 (1.0)	2 (3.3)	0.302	Pearson Chi-square
1	8 (7.6)	5 (8.3)		
2	22 (21.0)	19 (31.7)		
3	46 (43.8)	18 (30.0)		
4	28 (26.7)	16 (26.7)		
Perineural invasion (%)				
No	33 (47.8)	21 (45.7)	0.970	Pearson Chi-square
Yes	36 (52.2)	25 (54.3)		
Lymph-vascular invasion (%)				
No	49 (69.0)	38 (82.6)	0.153	Pearson Chi-square
Yes	22 (31.0)	8 (17.4)		

When P values < 0.05 was statistically significant

### Comparison of intestinal flora diversity in normal weight and overweight CRC patients

**Fig. 1:**
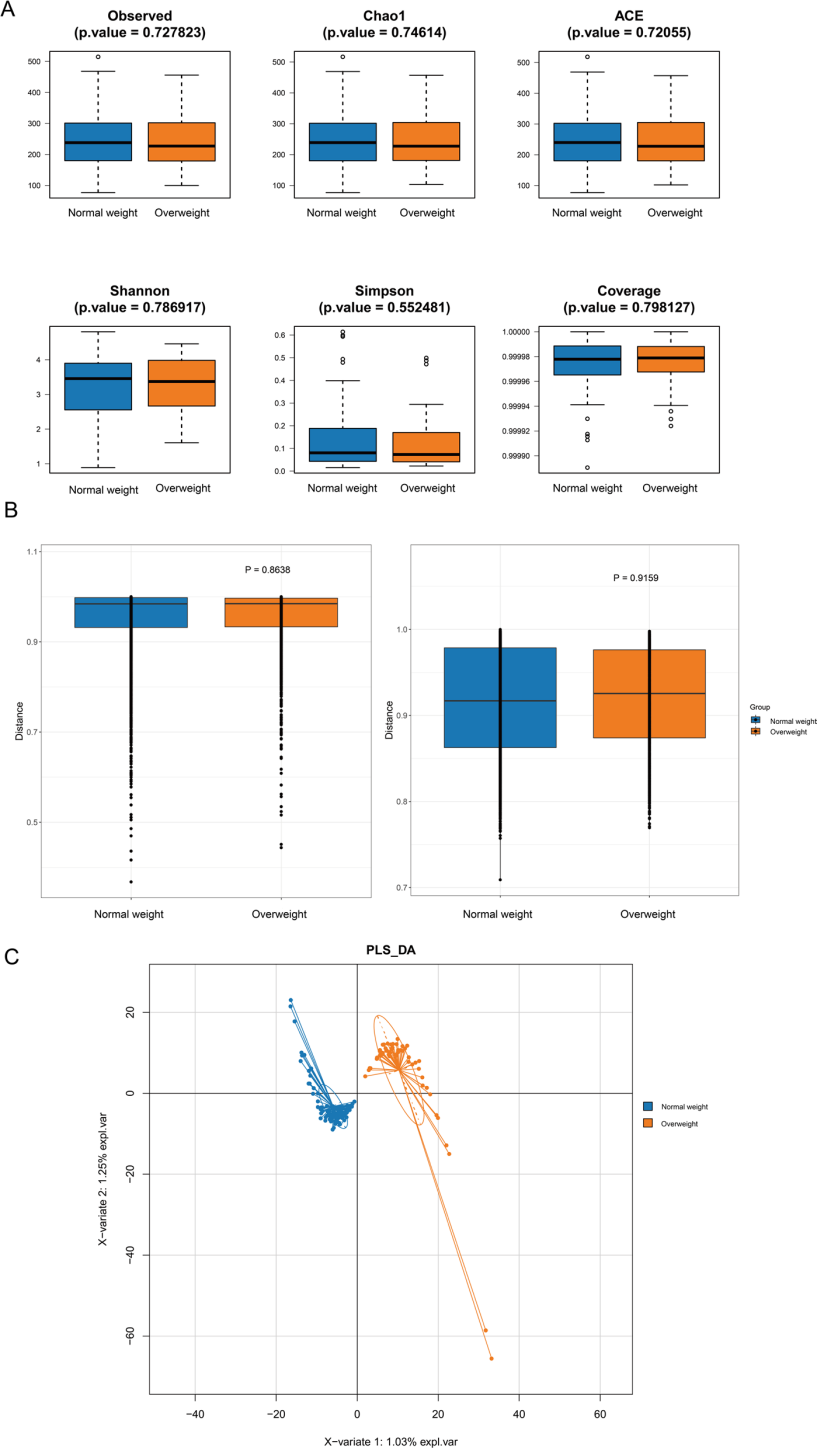
Comparison of intestinal flora diversity index of CRC patients in Overweight and Normal weight groups. **A** Comparison of α-diversity index of intestinal flora between Overweight group and Normal weight group of CRC patient group. **B** Comparison of β-diversity index of intestinal flora in Overweight group and Normal weight group of CRC patient group. The horizontal coordinates indicate the group, the vertical coordinates indicate the value of community diversity index of the samples in this group, and the color indicates the group. **C** PLS-DA analysis of intestinal flora of CRC patients in Overweight group and Normal weight group. Dots represent each sample, colors represent groups, scales on the horizontal and vertical axes represent relative distances to each sample, and X-variables 1 and 2 represent factors that influence changes in gut flora composition in CRC patients in Overweight group and Normal weight group, respectively

We used α-diversity versus β-diversity analyses to explain whether there were differences in intestinal flora diversity between overweight and normal weight group CRC patients. The results in Fig. 1A show that the differences in the six indices of α-diversity analysis between the two groups were not statistically significant (P > 0.05). The results of the β-diversity analysis of the two groups are shown in Fig. 1B, and the differences between the Bray–Curtis (P = 0.8638) and Jaccard (P = 0.9159) indices of the two groups were not statistically significant (see Additional file 7: Table S1 and Additional file 8: Table S2). Subsequently, we performed PLS-DA analysis, the results of which are shown in Fig. 1C, where normal weight group and overweight group patients can be distinguished into two different clusters. The above results were able to show that there was no significant difference in the overall level of intestinal flora diversity between the two groups, but there were still significant intergroup differences in the composition of the intestinal flora between the two groups.

### Identification of BMI-associated intestinal flora

**Fig. 2:**
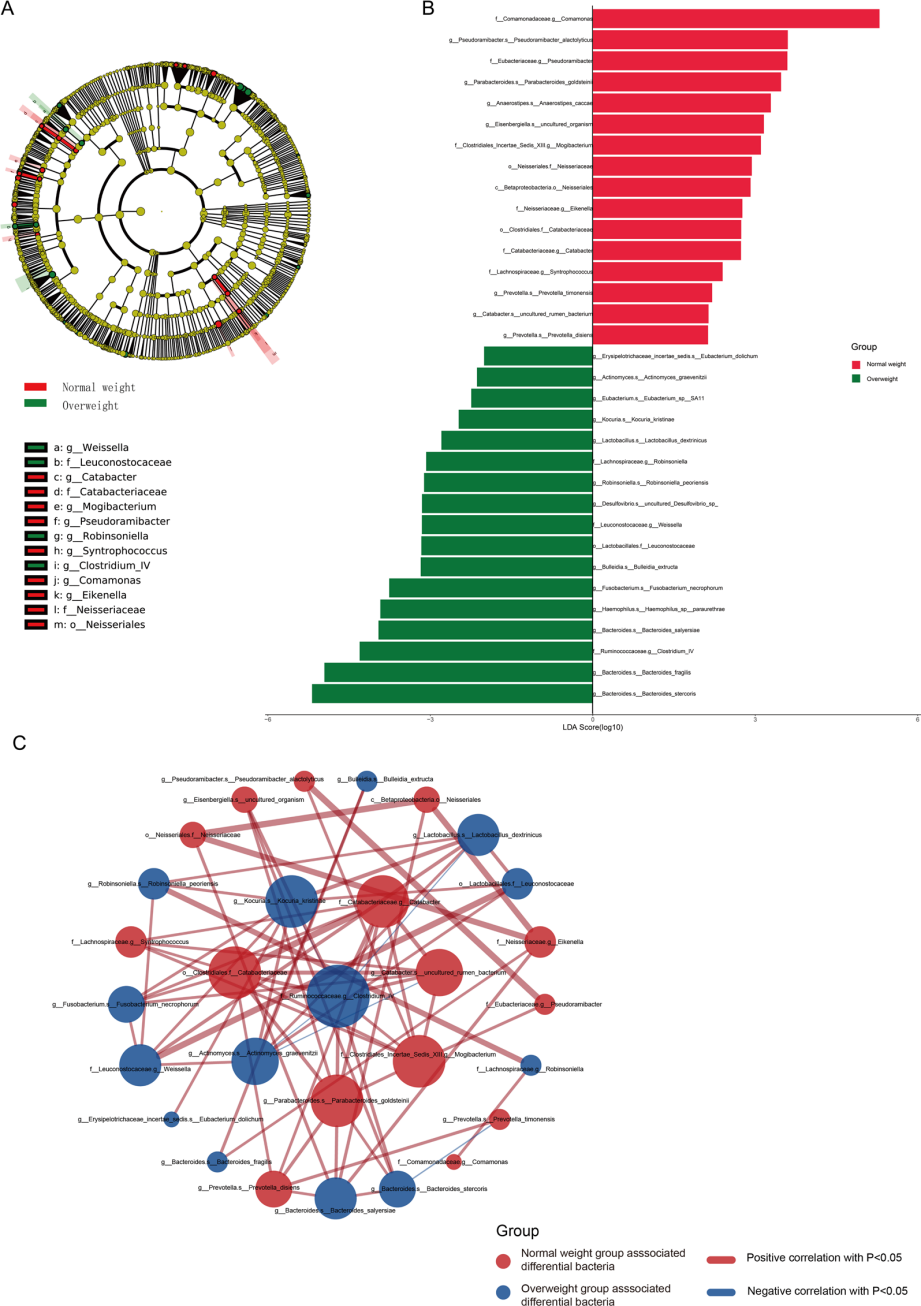
Analysis of intestinal flora difference between CRC patients in Overweight group and Normal weight group. **A** Cladogram of LEfSe analysis. Node size represents species abundance size and is proportional to species abundance size. Node colors represent groups, and yellow nodes in branches represent species that do not differ significantly in abundance between groups. Red nodes represent species with significantly higher abundance in group Normal weight, and green nodes represent species with significantly higher abundance in group Overweight. Nodes in each layer represent phyla/class/order/family/genus/species from the inside out, and species marker notes in each layer represent phyla/class/order/family/genus/species from the outside in. **B** LDA bar graph based on 16S rRNA gene sequencing. The color of the bar graph represents the group, the horizontal coordinate is the LDA score (processed by log10), the vertical coordinate represents the distinct species in the group with significantly higher abundance, and the length of the histogram represents the magnitude of the LDA score value. **C** Network diagram of BMI-related differential gut flora correlation. Each node represents each species, the node color represents the group, the node size represents the number of edges connected to the node, the larger the node, the more the number of edges connected to the node, the connection line represents a significant correlation between the two nodes, Spearman phase relationship value less than 0 (negative correlation) represents the blue line, and the number of nodes is the same. A Spearman correlation value greater than 0 (positive correlation) represents a red line, and the thicker the line, the greater the Spearman correlation coefficient between the two nodes

In order to identify differentially dominant intestinal flora in the intestinal flora of CRC patients in different BMI states, we performed LEfSe analyses on both overweight group and normal weight group CRC patients. A total of 33 statistically different intestinal flora were screened, of which 16 intestinal flora were significantly higher in abundance in the normal weight group than in the overweight group, and 17 intestinal flora were significantly higher in the overweight group than in the normal weight group (P < 0.05, see Additional file 9: Table S3, Fig. 2A, B), and in Fig. 2B, the LDA bar graphs show the LDA scores of LEfSe analyses for the two differentially dominant flora groups (scores after log10 treatment), with higher LDA scores indicating that the species was more able to discriminate between the two groups. In addition, to investigate the interactions of differential intestinal flora between normal weight group and overweight group CRC patients, we plotted the correlation between the two groups of dominant flora (Fig. 2C), where f__Ruminococcaceae.g__Clostridium_IV, g__Kocuria.s__Kocuria_kristinae, o__Clostridiales.f__Catabacteriaceae are the three bacteria most closely related to other dominant groups. Based on the above results, it can be found that the dominant flora between the two groups may be in competition with each other.

### Biofunctional prediction of intestinal flora in normal weight and overweight group CRC patients

**Fig. 3:**
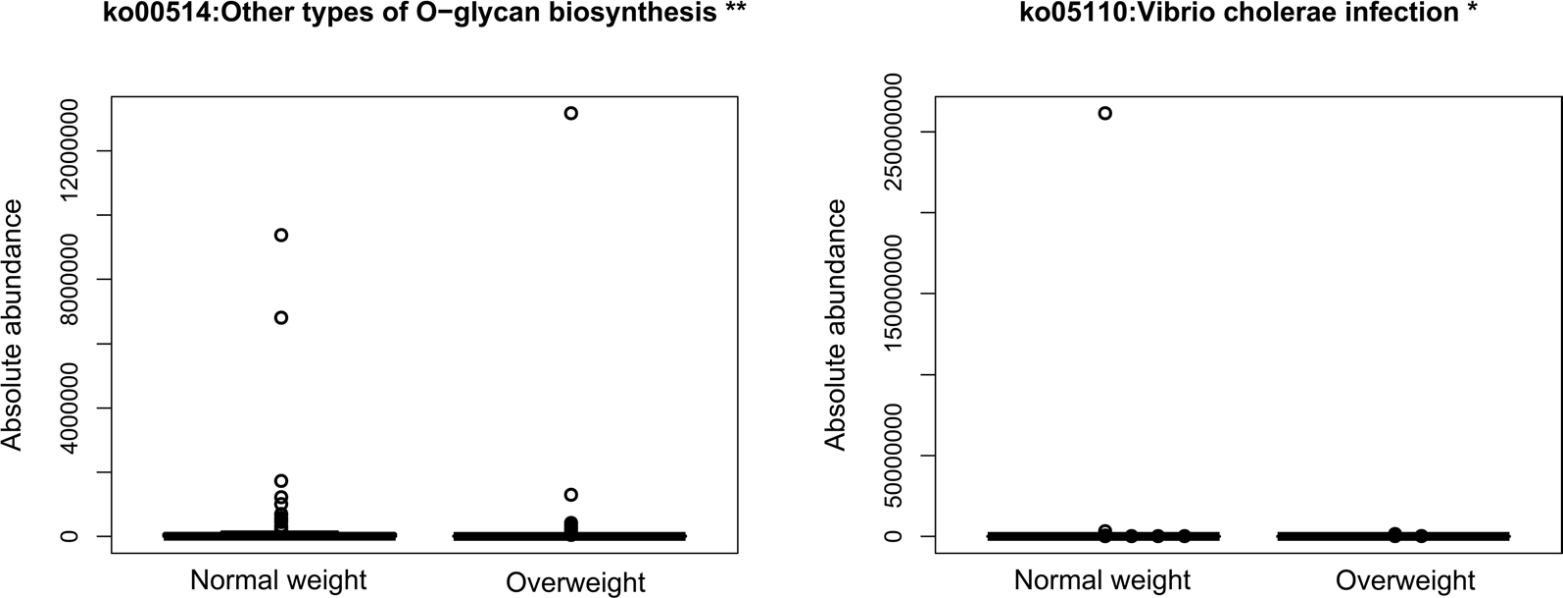
Box plot of KEGG functional abundance between the Overweight group and the Normal weight group of CRC cancer patients. The horizontal coordinate represents the grouping, and the vertical coordinate represents the predicted abundance. In each sample, the box edge represents the 25th to 75th percentile, the horizontal black line represents the median, and the extension line is equal to 1.5 times the maximum and minimum values between the quartiles

To investigate the biological pathways involved in gene enrichment in the intestinal flora of CRC patients with different BMI status, we screened for differences in KEGG pathways between normal weight and overweight patients using PICRUSt 2 software. A total of 179 differential KEGG pathways were screened, of which 2 pathways were statistically significantly different (P < 0.05), and these 2 up-regulated KEGG pathways were in the normal weight group, namely ko00514:Other types of O-glycan biosynthesis (P < 0.01) and ko05110:Vibrio cholerae infection (P < 0.05) (see Fig. 3, Additional file 10: Table S4). The above results suggest that the intestinal flora associated with BMI differences in CRC patients have different metabolic profiles.

### Relationship between BMI-associated intestinal flora and tumor-infiltrating immune cells

**Fig. 4:**
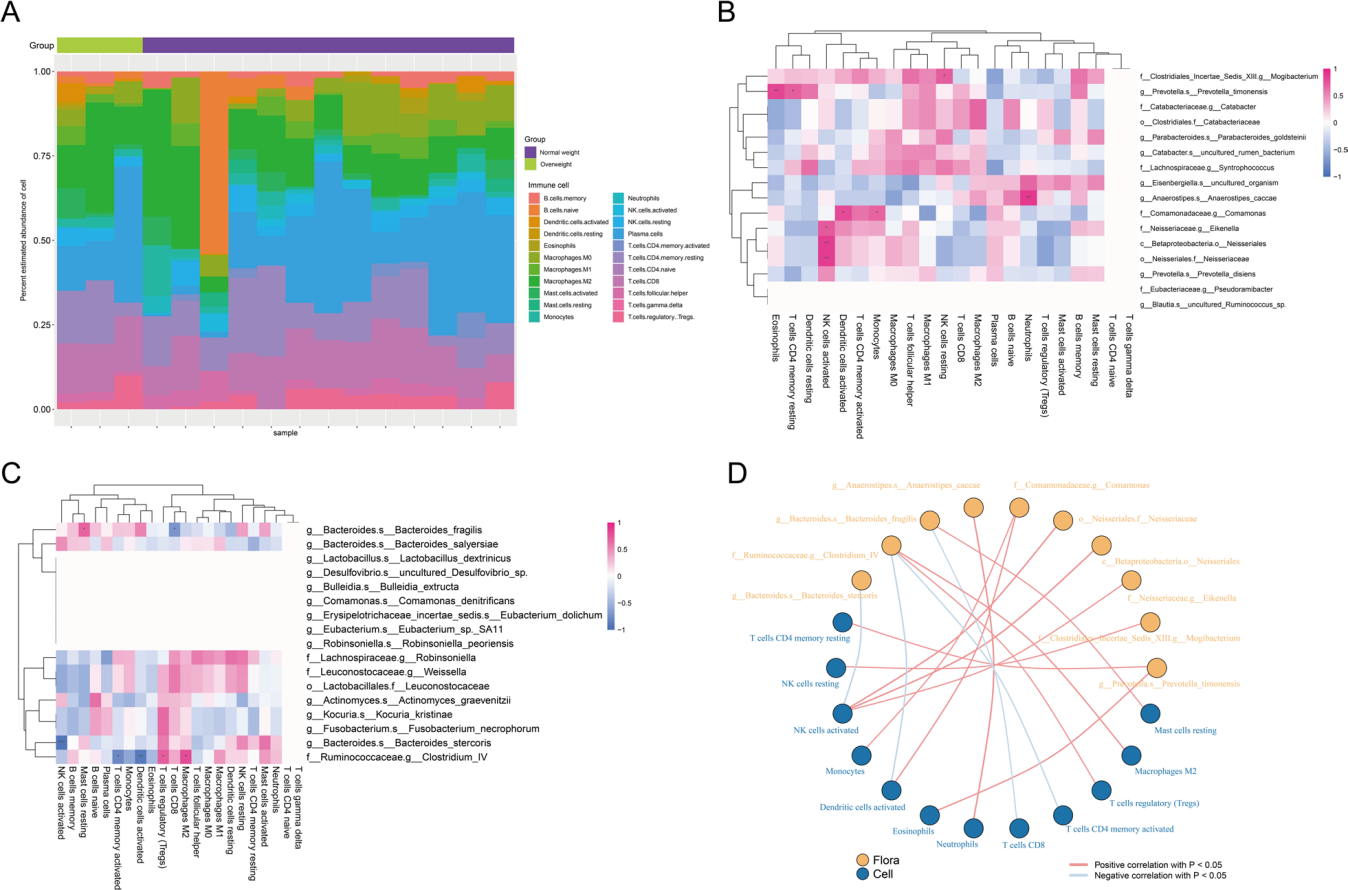
Relationship between BMI-associated intestinal flora and infiltrating immune cells in CRC. **A** Bar graph of relative abundance of immune cells in CRC patients grouped by BMI status. Each bar represents a sample, and each color in the graph corresponds to a type of immune cell. The ordinate is the relative abundance value of immune cells, and the sum of the relative abundance of all immune cells in a single sample is 1. **B** Heat maps of correlation between dominant colonies in the Normal weight group and tumor-infiltrating immune cells. **C** Heat maps of correlation between dominant colonies in the Overweight group and tumor-infiltrating immune cells. The horizontal coordinates are immune cells and the vertical coordinates are bacteria. Red indicates a positive correlation and blue indicates a negative correlation. The depth of the color indicates the size of the Pearson correlation coefficient, and the color from light to dark indicates the value of the phase relationship from small to large. The “*” in the graph represents the size of the P-value: No * for P-value ≥ 0.05, * for 0.01 ≤ P < 0.05, ** for 0.001 ≤ P < 0.01, *** for P < 0.001. **D** Network diagram of correlations between intestinal microbiota and immune cell differences associated with BMI. Nodes in different colors in the figure represent intestinal flora and immune cells respectively, and the connection lines between nodes indicate significant correlation between nodes. The blue line indicates that the Spearman correlation coefficient is less than 0 (negative correlation), while the red line indicates that the Spearman correlation coefficient is greater than 0 (positive correlation)

Tumour-infiltrating immune cells are an important component of the tumour immune microenvironment and can participate in the regulation of tumour-associated immune responses, both by inhibiting tumour growth and potentially promoting tumour metastasis and evasion of immune surveillance [29]. Therefore, tumour-infiltrating immune cells are regarded as a potential target for tumour immunotherapy. In order to explore the association between BMI-associated intestinal flora and tumour-infiltrating immune cells, we analysed the composition of 22 immune cells in 15 CRC patients with RNA sequencing information and BMI data, and plotted a bar graph of immune cell abundance (Fig. 4A), from which we can observe that the different BMI states unique features of the tumour immune microenvironment in CRC patients under different BMI. Afterwards, to explore the correlation between BMI-related differential intestinal flora and immune cells, we correlated the predominant intestinal flora of normal weight and overweight group CRC patients with 22 immune cells. In the group of normal weight CRC patients, c__Betaproteobacteria.o__Neisseriales, o__Neisseriales.f__Neisseriaceae, f__Neisseriaceae.g__Eikenella was significantly positively correlated with NK cells activated were significantly positively correlated, and g__Prevotella.s__Prevotella_timonensis was significantly positively correlated with T cells CD4 memory resting (Fig. 4B, D). In the group of overweight CRC patients, f__Ruminococcaceae.g__Clostridium_IV showed significant positive correlation with T cells regulatory and Macrophages M2, g__Bacteroides.s__Bacteroides_stercoris showed significant negative correlation with NK cells activated, and f__Ruminococcaceae.g__Clostridium_IV was significantly negatively correlated with Dendritic cells activated and T cells CD4 memory activated (Fig. 4C, D). The above results indicated that the BMI-related dominant intestinal flora of CRC patients were significantly correlated with a variety of tumour-infiltrating immune cells, suggesting that they may play a role in regulating the immune microenvironment of CRC.

### Correlation between BMI-related differences in intestinal flora and immune-related genes

**Fig. 5:**
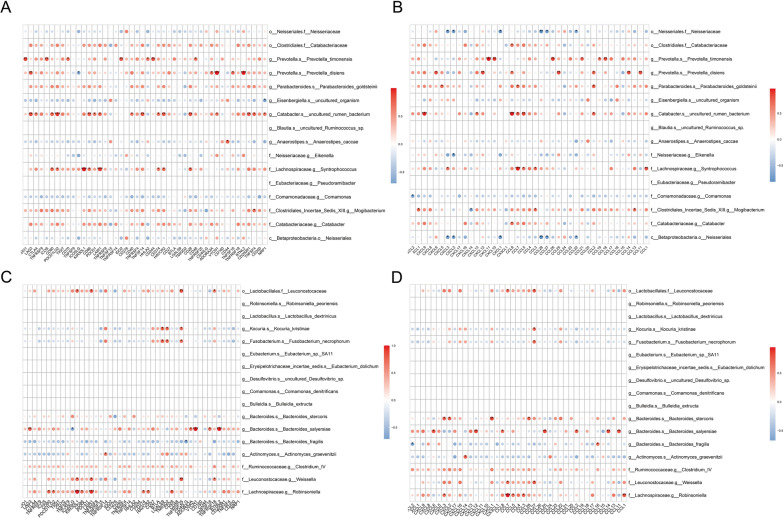
Correlation between BMI-related differences in intestinal flora and immune-related genes. **A** Heat map of correlation between the dominant flora and checkpoints in the Normal weight group. **B** Heat map of the correlation between the dominant flora and chemokines in the Normal weight group. **C** Heat map of correlation between the dominant flora and checkpoints in the Overweight group. **D** Heat map of the correlation between the dominant flora and chemokines in the Overweight group. In the figure, the horizontal coordinate is the immune-related genes, and the vertical coordinate is the bacteria. In the figure, the red indicates the positive correlation, and the blue indicates the negative correlation. The color depth indicates the size of the Pearson correlation coefficient, and the color from light to dark indicates the value of the phase relationship from small to large. The “*” in the graph represents the size of the P-value: No * for P-value ≥ 0.05, * for 0.01 ≤ P < 0.05, ** for 0.001 ≤ P < 0.01, *** for P < 0.001

The human immune system is closely linked to tumour progression, and in order to investigate the relationship between BMI-associated differential intestinal flora and organismal immunity, we analysed the correlation between BMI-associated intestinal flora and immune-related genes. Among the normal weight group differential flora, g__Catabacter.s__uncultured_rumen_bacterium, f__Lachnospiraceae.g__Syntrophococcus and g__Prevotella.s__Prevotella_disiens with multiple immune checkpoints (BTNL2, CD86 and LAG3, among others) (Fig. 5A), immune-activating genes (CD28, ENTPD1 and CD86, among others) (see Additional file 1: Figure S1), immune-suppressing genes (HAVCR2, PDCD1LG2, LAG3 and CD274, among others) (see Additional file 2: Figure S2), chemokines (CCL8, CCL7, CXCL9, among others) (Fig. 5B), and chemokine receptors (CCR2, CCR1, and XCR1, etc.) (see Additional file 3: Figure S3) showed significant positive correlations. Among the overweight groups of differential bacteria, o__Lactobacillales.f__Leuconostocaceae, f__Lachnospiraceae.g__Robinsonella and g__Bacteroides.s__Bacteroides_salyersiae were associated with multiple immune checkpoints (KIR3DL1, LAIR1, TNFRSF25, etc.) (Fig. 5C), immune-activating genes (TNFRSF25, TNFRSF17, BTNL2, etc.) (see Additional file 4: Figure S4), immune-suppressing genes (KIR2DL1, VTCN1, LAG3, etc.) (see Additional file 5: Figure S5), chemokines (CCL7, CXCL12, CCL25, etc.) (Fig. 5D), and chemokine receptors (CCR2, XCR1, etc.) (see Additional file 6: Figure S6) showed significant positive correlations. The above results suggest that BMI-related differences in intestinal flora may influence the expression of immune-related genes.

### Identification of BMI-related differential functional pathways and correlation of differential pathways with differential intestinal flora

**Fig. 6:**
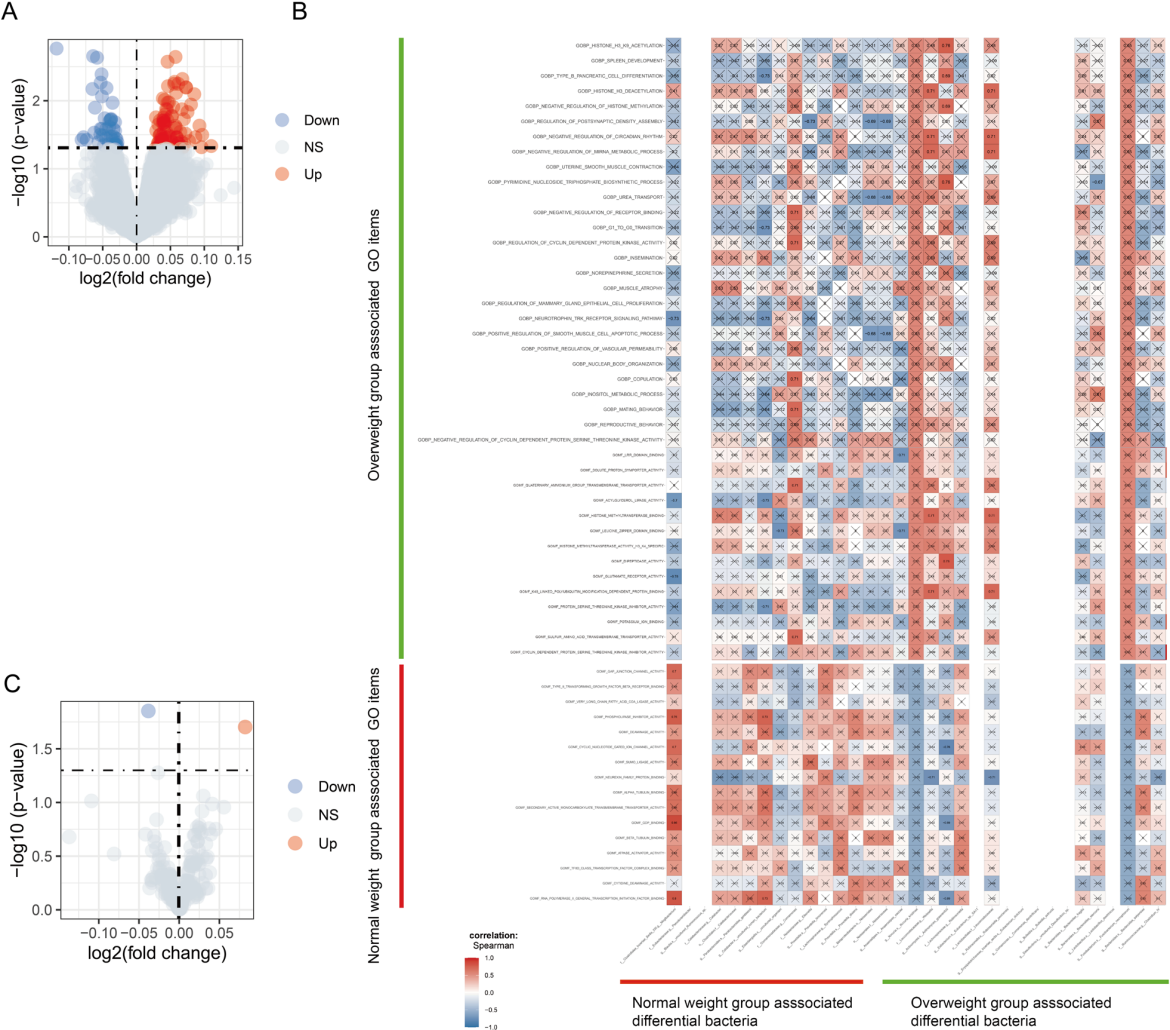
Identification of BMI-related differential pathways and their correlation with BMI-related differential intestinal flora. **A** GO volcano plot of the associated differential expression in the Normal weight group versus Overweight group. **B** KEGG volcano plot of the associated differential expression in the Normal weight group versus Overweight group. The horizontal coordinates indicate log 2 (fold change). The farther the point is from the center, the larger the multiple of the difference; The vertical coordinates represent −log 10 (P-value). The closer the point is to the top, the more significant the difference in expression. Each dot represents the differentially expressed gene detected. Red indicates upregulated genes. Blue indicates down-regulated genes. Gray indicates no differential genes. **C** BMI-related differences Correlation graph of intestinal flora with BMI-related differences in BP and MF. The horizontal coordinate of the graph is bacteria. The vertical coordinates are the GOMF items. In this figure, red indicates positive correlation, blue indicates negative correlation, color depth indicates the size of Spearman correlation coefficient, and color from light to dark indicates the value of phase relationship from small to large. The symbol “×” in the figure represents the size of the P-value: the presence of “×” means the P-value ≥ 0.05, and the absence of “×” means P 0.05

In order to explore the differential functional pathways in normal weight and overweight group CRC patients and to investigate the correlation between the differential intestinal flora of the two groups and these pathways, we used the ssGSEA methodology to transform the gene expression matrix obtained by RNA sequencing from tumour tissue samples of nine CRC patients and the species abundance matrix obtained by 16S rRNA sequencing of the intestinal flora into the corresponding scoring matrices and scored them via the KEGG and GO analyses for scoring. The GO analyses mainly included three main domains: cellular component (CC), molecular function (MF), and biological process (BP). We then analysed the differences in KEGG and GO pathways between the two groups. In normal weight group, we identified a total of 86 significantly up-regulated GO pathways [GOBP_PROTEIN_DENEDDYLATION (logFC = 0.057, P = 0.002) and GOBP_COPPER_ION_TRANSPORT (logFC = 0.027, P = 0.048), etc.] and 1 significantly upregulated KEGG pathway [KEGG_PROTEIN_EXPORT (logFC = 0.082, P = 0.019)]. In the overweight group, we identified a total of 43 significantly up-regulated GO pathways [GOBP_UREA_TRANSPORT (logFC = − 0.049, P = 0.032) and GOBP_SPLEEN_DEVELOPMENT (logFC = − 0.031, P = 0.045), etc.] and 1 significantly down-regulated KEGG pathway [KEGG_TYPE_II_DIABETES_MELLITUS (logFC = -0.038, P = 0.014)] (shown in Fig. 6A, B). Detailed lists of KEGG-enriched pathways and GO-enriched items are shown in Additional file 11: Table S5 and Additional file 12: Table S6, respectively. The above results suggest that different biological functions exist in normal weight and overweight group CRC patients. Subsequently, we investigated the relationship between BP items, MF items and KEGG pathways and BMI-related differences in intestinal flora in both groups, and found that some of the intestinal flora were significantly correlated with some biological pathways. For example, f__Clostridiales_Incertae_Sedis_XIII.g__Mogibacterium and GOBP_PURINE_NUCLEOSIDE_BIOSYNTHETIC_PROCESS showed significant positive correlation (r = 0.71, P = 0.031) (Fig. 6C, Additional file 13: Table S7). f__Neisseriaceae.g__Eikenella and KEGG_PROTEIN_EXPORT showed significant positive correlation (r = 0.75, P = 0.019) (see Additional file 14: Table S8). The above results suggest that differential intestinal flora associated with BMI may be able to influence CRC patients with different BMI status through potential biological functional pathways.

## Discussion

In the context of modern rapidly developing societies, overweight has gradually become a global health problem due to changes in people's living, eating and exercise habits, and overweight is often accompanied by a variety of cardiovascular diseases, metabolic diseases and cancers [3, 30]. The number of overweight adults in the US and UK is expected to increase by 65 million and 11 million, respectively, while the number of overweight-related cancers will increase by 492,000–669,000 cases by 2030 [31], yet so far, there are few studies on the correlation between different BMI statuses, intestinal flora and CRC. In this study, we investigated the species diversity and relative abundance of intestinal flora in normal weight and overweight CRC patients based on 16S rRNA sequencing and searched for potential biological functional pathways associated with BMI. We first performed α-diversity and β-diversity analyses on the stool samples of the two groups, and the results showed that the results of both diversity analyses suggested that the differences were not statistically significant (as shown in Fig. 1A, B), which indicated that the diversity of the intestinal flora of the two groups was not significantly different at the overall level. A study using 16S rRNA sequencing to assess the intestinal flora differences between 36 CRC patients and 38 healthy individuals showed that the intestinal flora α-diversity Chao1 index and Shannon index were significantly higher in CRC patients than in healthy controls [32]. Another study used 16S rRNA sequencing to compare the intestinal microbial community structure of healthy control group, polyp patients, and CRC patients, and found that there was no significant difference in intestinal microbial composition between CRC patients and polyp patients, and the ACE index of intestinal flora α-diversity in CRC patients was significantly lower than that in healthy control group [33]. Another researcher compared the differences in intestinal flora in CRC patients with different BMI status, and showed that compared to normal weight healthy people, CRC patients with BMI > 25 kg/m^2^ showed significant enrichment of Clostridium in their intestinal flora [32]. Therefore, we hypothesised that the occurrence of CRC may be closely related to the intestinal flora, and at the same time, the occurrence of CRC and the composition of the intestinal flora may also be affected by the BMI status. Subsequently, we performed a least squares discriminant analysis (PLS-DA) on the two groups, which showed that the intestinal flora of the normal weight and overweight groups of patients could be distinguished into two different clusters (as shown in Fig. 1C), suggesting that there was a significant difference in the species composition of the two groups. Therefore, we further identified the species of the flora that differed significantly between the two groups and analysed the relationship between the differential flora, BMI and CRC.

We identified a total of 33 differential intestinal flora species associated with BMI by LEfSe analysis, of which Actinobacteria, Desulfovibrio, Bacteroides were significantly enriched in the intestinal microecology of overweight CRC patients. Desulfovibrio is a Gram-negative bacterium, and it has now been shown that Desulfovibrio is able to reduce intestinal sulphate to hydrogen sulphide, and that hydrogen sulphide produced by the bacterium induces DNA damage in colonic epithelial cells and promotes the proliferation of colorectal cancer cells [34, 35]. A study that collected follow-up data from 51,529 men to determine the association between faecal sulfur-metabolizing bacteria and CRC risk showed that long-term adherence to dietary patterns associated with faecal sulfur-metabolizing bacteria was associated with an increased risk of distal CRC [36]. In addition, hydrogen sulphide produced by Desulfovibrio reduces disulphide bonds thereby disrupting the intestinal barrier, inducing elevated levels of inflammatory and pro-metastatic cytokines, and increasing the metastatic potential of CRC by remodelling the extracellular matrix and forming a metastatic microenvironment [37]. This suggests that Desulfovibrio might play a potential role in the onset, progression and metastasis of CRC. Andrew M Thomas et al. used 16S rRNA sequencing to compare the intestinal flora of 18 CRC patients with 18 healthy individuals. Compared with healthy individuals, Bacteroides fragilis in the Bacteroides genus showed significant enrichment in CRC patients [38], furthermore, the increased abundance of Bacteroides fragilis in the intestinal microenvironment was positively correlated with overweight [39], which is largely in consistent with our findings. Actinobacillus have also been shown to coexist with a variety of CRC-associated intestinal flora, including Bacteroides. Also, Actinobacillus co-localise with tumour-associated fibroblasts in CRC, while being able to activate the TLR2/NF-κB pathway, thereby reducing CD8 + T lymphocyte infiltration in the CRC microenvironment [40]. In summary, we hypothesise that Actinobacillus, Desulfovibrio, and Bacteroides are closely associated with overweight CRC patients, and may serve as potential biological markers of CRC associated with BMI status.

In the normal weight group, Anaerostipes and Prevotella showed significant enrichment in intestinal flora compared with overweight. Anaerostipes is a Gram-positive anaerobic bacterium colonising the human gut, which metabolises myo-inositol into short-chain fatty acids(SCFA) such as propionate and butyrate [41], which not only provide nutrients to the intestinal epithelial cells, but also inhibit the expression of pro-inflammatory cytokines, including IL-6, TNF-α and IL-17, and ameliorate the chronic inflammation in the intestinal tract [42]. It has been found that the abundance of SCFA and SCFA-producing bacteria is significantly reduced in the intestinal microecology of CRC patients, and that supplementation with beneficial SCFA-producing bacteria may inhibit the development of intestinal tumours [43]. Prevotella is an important member of the human intestinal flora, a study collected fecal samples from 89 CRC patients and 161 healthy individuals, and used metagenomics to study the relationship between intestinal flora and CRC. The results showed that the relative abundance of Prevotella in the gut microbiota of CRC patients was significantly reduced [44], while Prevotella appeared enriched in healthy controls [45], suggesting that Prevotella enrichment might be a protective factor against CRC. In addition, Prevotella has the ability to ferment and degrade complex carbohydrates such as cellulose [46], and there is a significant correlation between the enrichment of this colony and a high-fibre, low-fat diet [47], which may explain the enrichment of Prevotella in the normal weight group that occurred in our study. Meanwhile, the high-fibre, low-fat Mediterranean dietary pattern, which helps to maintain a normal BMI status, is also effective in preventing CRC [48].

We used PICRUSt 2 software to predict KEGG differential pathways in the intestinal flora of normal weight versus overweight group CRC patients. The results showed that ko00514: Other types of O-glycan biosynthesis were significantly enriched in overweight group compared to normal weight group. o-glycan is a glycoprotein assembled by the sequential action of several specific membrane-bound glycosyl groups, O-acyl groups, and sulphotransferases in a highly regulated manner [49], which has a complex structure and can participate in a variety of biological processes related to tumourigenesis and progression [50]. Many studies have shown that aryl glycans accumulate in colorectal cancer cells after exposure of colorectal cancer cells to O-glycan inhibitors, thereby inhibiting cancer cell proliferation and inducing apoptosis [51–53]. In addition, some O-glycan, such as Tn antigen, has been shown to be widely expressed in various cancers, including CRC [54], and can be used as a biomarker target for human cancers [55]. Meanwhile, it has been found that Tn antigen-positive CRC cells exhibit enhanced metastatic ability in both in vivo and in vitro experiments [56]. Taken together, we speculate that patients with overweight group CRC may be involved in CRC development and metastasis by enhancing the role of O-glycan-related biological pathways.

We explored the correlation of BMI-associated differential flora with immune cells and immune-related genes and found that in overweight groups of CRC patients Clostridium IV had significant positive correlations with Macrophages M2 and T cells regulatory (Tregs) and significant negative correlations with Dendritic cells activated and T cells CD4 memory activated (shown in Fig. 4C) and a significant negative correlation with the immune activation gene ULBP1 (see Additional file 4: Figure S4). It has been found that CRC patients showed a significant enrichment of Clostridium IV in their intestinal flora compared to healthy controls [57, 58]. Notably, a significant stepwise increase in the abundance of Clostridium spp. was observed in colorectal adenomas, early-stage CRC, and late-stage CRC [59], suggesting that Clostridium spp. are enriched in the early stages of colorectal tumourigenesis. Clostridium IV is able to participate in the modulation of the intestinal immune microenvironment, and an animal study found that Clostridium IV colonisation of the mouse intestine significantly up-regulated the level of intestinal Tregs [60], Clostridium IV was able to provide bacterial antigens and a transforming growth factor-β (TGF-β)-rich environment, which promoted the proliferation and differentiation of Tregs [61], and Tregs expressed the immune checkpoint receptor for cytotoxic T lymphocyte-associated antigen-4 (CTLA-4), which promotes tumour immune tolerance [62]. Macrophages M2 is able to secrete anti-inflammatory factors such as IL-10 and IL-1β involved in angiogenesis and tumourigenesis and progression [63]. UL-16 binding protein 1 (ULBP1) is a natural killer cell that can activate the natural killer cells and plays an important role in immune regulatory protein [64]. Currently, it has been found that ULBP1 activates NKG2D receptors on the surface of some T cells and natural killer cells to mediate anti-tumour immunity [65]. Therefore, we speculate that Clostridium IV is involved in the development and progression of CRC by altering the intestinal immune microenvironment.

Subsequently, we found a significant positive correlation (Spearman r = 0.71, P = 0.033) between the genus Comamonas and GOBP_REGULATION_OF_CYCLIN_DEPENDENT_PROTEIN_KINASE_ACTIVITY (Fig. 6C). Cell cycle protein-dependent kinases (CDKs) are protein kinases that can participate in the regulation of various stages of the cell cycle and play an important role in the regulation of cell division [66]. In the normal cell division pathway and in the absence of exogenous DNA damage, the checkpoint kinases ATR and CHK1 are able to control genome integrity by inhibiting the activity of CDKs during DNA synthesis, and aberrant activation of CDKs leads to DNA breakage damage [67–69], a mechanism that is not yet fully understood. Following mutation or other inactivation of the checkpoint kinases ATR and CHK1 during tumourigenesis, these replication-associated DNA lesions may lead to loss of genomic integrity and cancer development [70]. A large number of studies have found that several inhibitors of CDKs, including salicylic acid metabolites and flavonoid metabolites, have favourable anticancer activity in CRC cells [71–73]. In addition, it has also been shown that the levels of CDKs were significantly elevated in CRC tissues, while overexpression of CDKs in CRC tissues correlated significantly with nodal metastatic status and histological subtype [74]. The genus Comamonas releases a variety of virulence factors such as haemolysins and metabolic enzymes, and it is an opportunistic pathogen in humans [75]. There have been case reports that the genus Comamonas is susceptible to cause infection in patients with underlying diseases such as malignancy and liver disease [76]. In addition, intestinal mucosal microbiota samples from 29 CRC patients and 29 age-matched healthy controls were collected for metagenomic studies. Comamonas was significantly enriched in intestinal flora in CRC group compared with the control group [77]. We speculate that the harmful metabolites released by Comamonas along with a variety of virulence factors might be able to affect the expression of CDKs, which indirectly participate in the regulation of the cell cycle and lead to the development of CRC. However, there is a lack of relevant studies, and our speculation remains open to further research.

In this study, based on 16S rRNA sequencing and transcriptome sequencing, the sequencing data of intestinal flora and tumor tissues under different BMI states were obtained, and the differential flora under different BMI states was obtained through microbial diversity analysis. CIBERSORT algorithm was used to study the correlation between different intestinal flora and immune cells and immune-related genes in different BMI states. Finally, BMI-related differential functional pathways were identified and the correlation between these pathways and differential intestinal flora was analyzed. Compared with 16S rRNA sequencing or transcriptome sequencing alone, this study combined the two methods and used multiple analysis algorithms to better elucidate the relationship between intestinal flora and CRC under different BMI states, and reveal the characteristics of tumor immune microenvironment under different BMI states. This study also has certain limitations. First, compared with metagenomic sequencing, 16S rRNA gene sequencing cannot annotate some species at the species level, and the depth of species identification by 16S rRNA gene sequencing is relatively shallow. Metagenomic sequencing can conduct high-throughput sequencing of the total DNA of all microorganisms to obtain more detailed microbial population structure, gene functional activity, and the relationship between microorganisms and microbes and between microorganisms and the environment [78]. Secondly, this study did not include healthy people as the control group, so it was impossible to compare the differences in intestinal flora between CRC patients and healthy people under different BMI status. In addition, fecal samples are susceptible to factors such as food intake, digestive process and intestinal transport time, and cannot well reflect the overall picture and subtle changes of intestinal flora [79]. This study still needs to further improve the correlation experiment between gene targets and overweight CRC patients to verify the accuracy of the results. Subsequently, it was considered to collect more CRC lesions and surrounding normal mucosal tissues for analysis, so as to obtain more accurate microflora information, and help to study the microflora structure and immune microenvironment changes of lesions and normal tissues.

## Conclusion

There are a total of 33 types of intestinal flora with significant differences between normal weight group and overweight group CRC patients. Among them, Actinomyces, Desulphurvibrio and Bacteroides are significantly enriched in the intestinal flora of overweight CRC patients, while Anaerostipes and Prevotella are significantly enriched in the intestinal flora of CRC patients in the normal weight group. ko00514: Other types of O-glycan biosynthesis pathway are significantly enriched in overweight group, which may be related to the occurrence and metastasis of CRC. A significant positive correlation is found between Clostridium IV and Macrophages M2 and T cells regulatory (Tregs) in overweight group CRC patients. There was a significant negative correlation with Dendritic cells activated and T cells CD4 memory activated, suggesting that some intestinal flora may regulate immune cell infiltration and immune gene expression to affect the biological process of CRC. But further experiments are needed to confirm this. In this study, the relationship between BMI, CRC and intestinal flora was discussed from the perspectives of flora diversity, immune-related factors and biological functional pathways, which helps to explain the mechanism of CRC occurrence and progression.

### Supplementary Information

**Additional file 1: Figure S1.** Heat map of correlation between dominant bacteria and immune activation genes in the Normal weight group.

**Additional file 2: Figure S2.** Heat map of correlation between dominant bacteria and immunosuppressive genes in the Normal weight group.

**Additional file 3: Figure S3. **Heat map of correlation between dominant bacteria and chemokine receptors in the Normal weight group.

**Additional file 4: Figure S4.** Heat map of correlation between dominant bacteria and immune activation genes in the Overweight group.

**Additional file 5: Figure S5.** Heat map of correlation between dominant bacteria and immunosuppressive genes in the Overweight group.

**Additional file 6: Figure S6.** Heat map of correlation between dominant bacteria and chemokine receptors in the Overweight group. Horizontal coordinate is gene, vertical coordinate is colony, red represents positive correlation, blue represents negative correlation, color depth represents Pearson correlation coefficient size, color from light to dark indicates Pearson correlation coefficient value from small to large. The "*" in the graph represents the size of the p-value: No * for P-value ≥ 0.05, * for 0.01 ≤ P < 0.05, ** for 0.001 ≤ P < 0.01, *** for P < 0.001.

**Additional file 7: Table S1.** ADONIS test for Bray Distance of intestinal flora in CRC patients in the Overweight and Normal weight groups.

**Additional file 8: Table S2.** ADONIS test for Jaccard Distance of intestinal flora in CRC patients in the Overweight and Normal weight groups. Group row: between-group statistics; Df: degrees of freedom, between-groups degrees of freedom as number of groups-1, within-groups degrees of freedom as total number of samples—number of groups; Residuals row: statistical information within the group; Total row: intergroup + intragroup statistics; Sums Of Sqs: sum of squares of deviation; Mean Sqs: Mean square, the ratio of the sum of squared deviations to the degrees of freedom, i.e. Sums Of Sqs/Df; F.Model: F test value, i.e. mean square between groups/mean square within groups; R2: the proportion of the sum of squares of deviations between groups and within groups to the sum of squares of total deviations, indicating the degree of explanation for inter-sample differences. The larger R2 indicates the higher degree of explanation for inter-sample differences. Pr(> F): statistically significant P-values derived from replacement tests, Pr < 0.05 was considered statistically significant.

**Additional file 9: Table S3.** Results of LEfSe analysis of intestinal flora between Normal weight and Overweight groups CRC patients. Taxonomy: BMI related intestinal flora information; Group: group with significant abundance of differential species; LDA: effect value of BMI-associated gut flora after log10 treatment; species with LDA scores (log10) greater than 2 and p-values less than 0.05 are shown in Table.

**Additional file 10: Table S4.** KEGG functional pathways in the intestinal microbiome of CRC patients in the Overweight and Normal weight. KEGG_Pathway: KEGG pathway; Mean In Normal weight: the predicted abundance value of this pathway in each sample in the Normal weight group; Mean In Overweight: the predicted abundance value of this pathway in each sample in the Overweight group. Statistically significant when p-value is less than 0.05.

**Additional file 11: Table S5.** List of differential KEGG pathways of CRC patients stratified by BMI condition. KEGG pathway: enriched KEGG pathway. logFC: FC represents the folding change, that is, the ratio of the expression of the Overweight group and the Normal weight group. The logarithm is taken as the base of 2. Statistically significant when p-value is less than 0.05.

**Additional file 12: Table S6.** List of differential GO items of CRC patients stratified by BMI condition. GO items: Enriched GO entries. LogFC: FC represents the folding change, that is, the ratio of the expression of the Overweight group and the Normal weight group. The logarithm is taken as the base of 2. Statistically significant when p-value is less than 0.05.

**Additional file 13: Table S7. **Correlation between BMI-associated enrichment of GO and BMI-associated dominance of intestinal flora.

**Additional file 14: Table S8.** Association between BMI-associated KEGG enrichment pathways and BMI-associated dominant intestinal flora. The r.value is the Spearman correlation coefficient value. P-value less than 0.05 is statistically significant.

## Data Availability

The original contributions presented in the study are included in the article material, further inquiries can be directed to the corresponding authors.
