# Supplementary figures and images for: Analysis of differences in intestinal flora associated with different BMI status in colorectal cancer patients
Source: J Transl Med. 2024 Feb 9;22:142. doi: 10.1186/s12967-024-04903-7 (PMC10854193; doi:10.1186/s12967-024-04903-7)

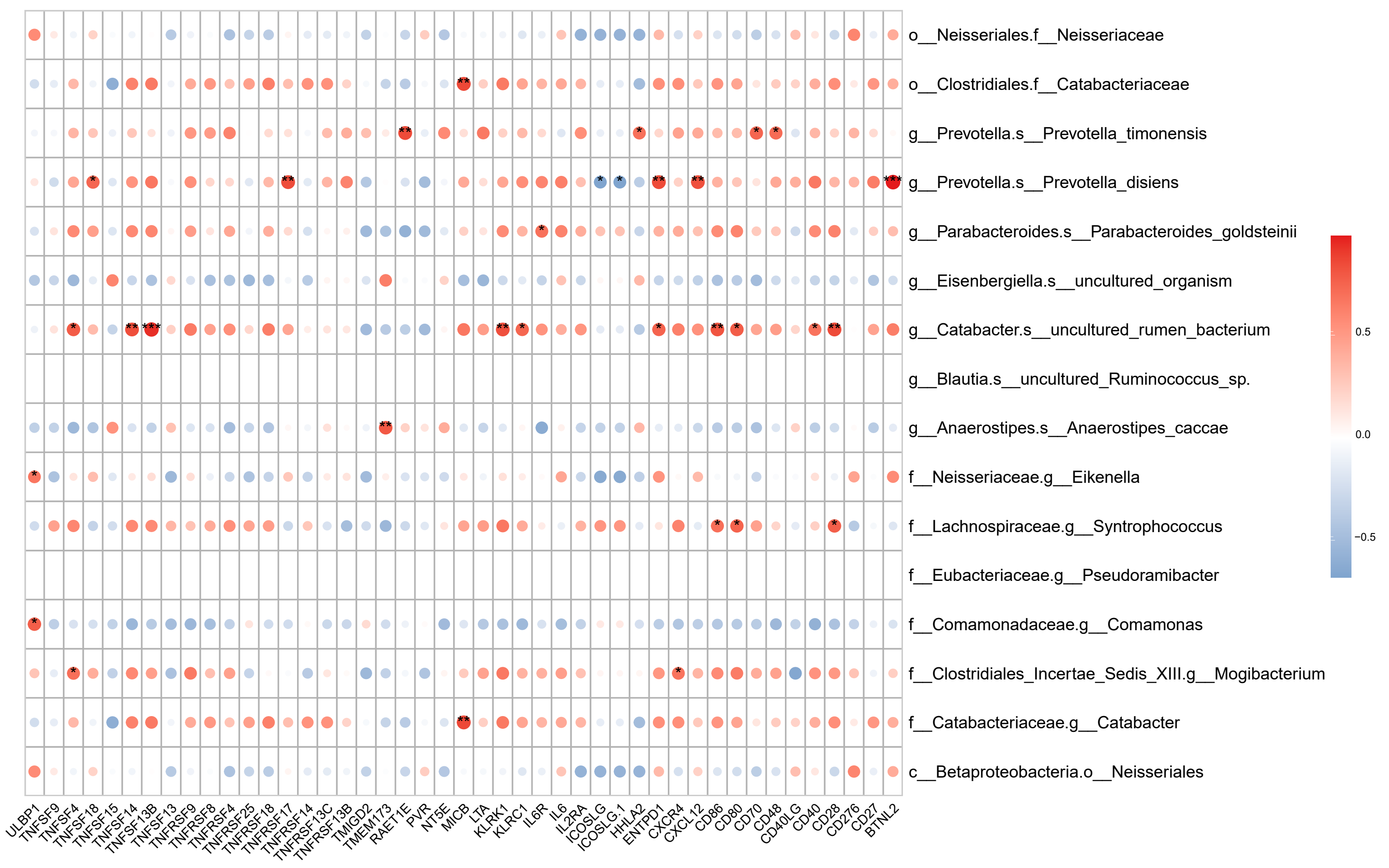

Supplement: Supplementary file 1 — Additional file 1: Figure S1. Heat map of correlation between dominant bacteria and immune activation genes in the Normal weight group. [file 12967_2024_4903_MOESM1_ESM.pdf]

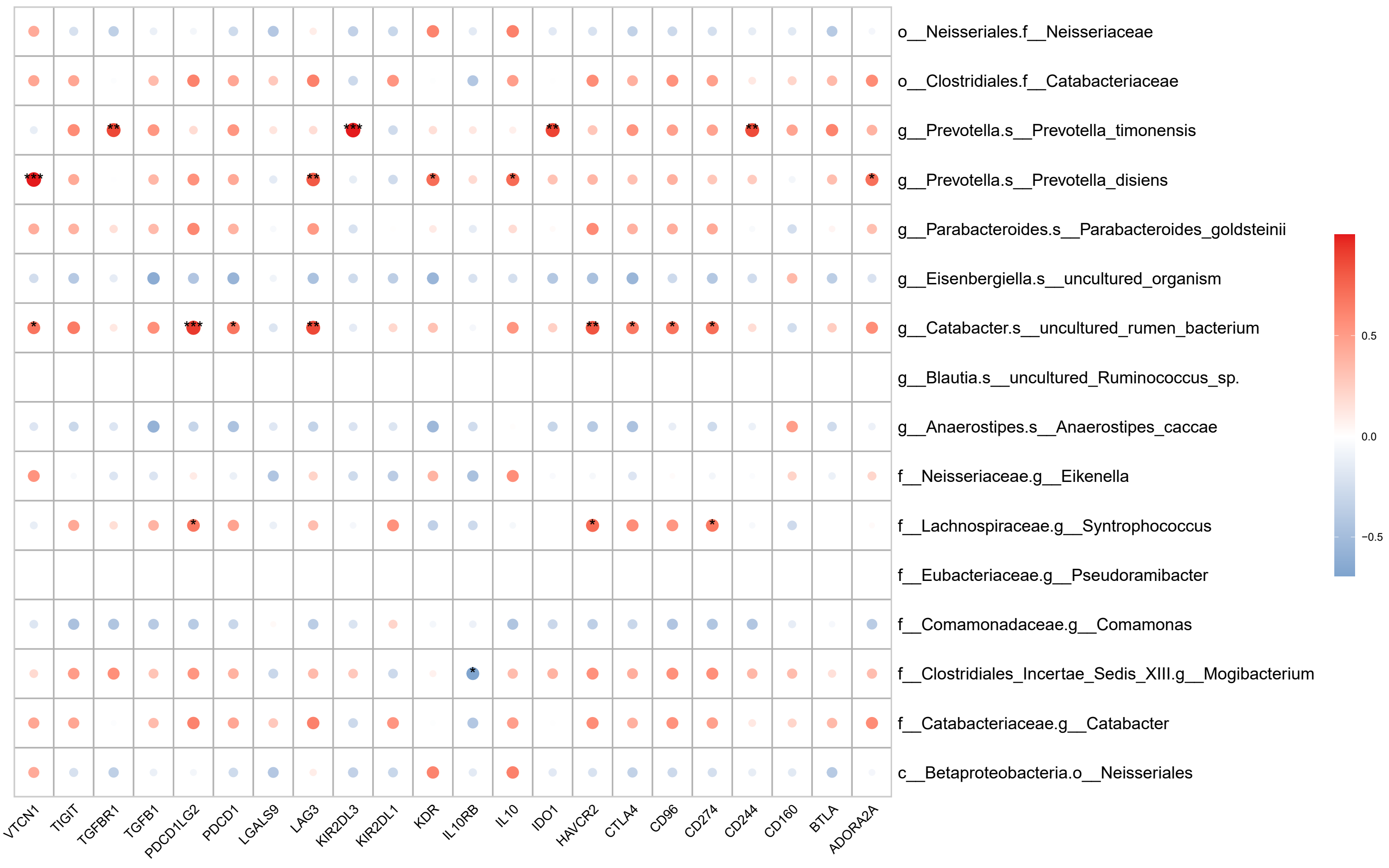

Supplement: Supplementary file 2 — Additional file 2: Figure S2. Heat map of correlation between dominant bacteria and immunosuppressive genes in the Normal weight group. [file 12967_2024_4903_MOESM2_ESM.pdf]

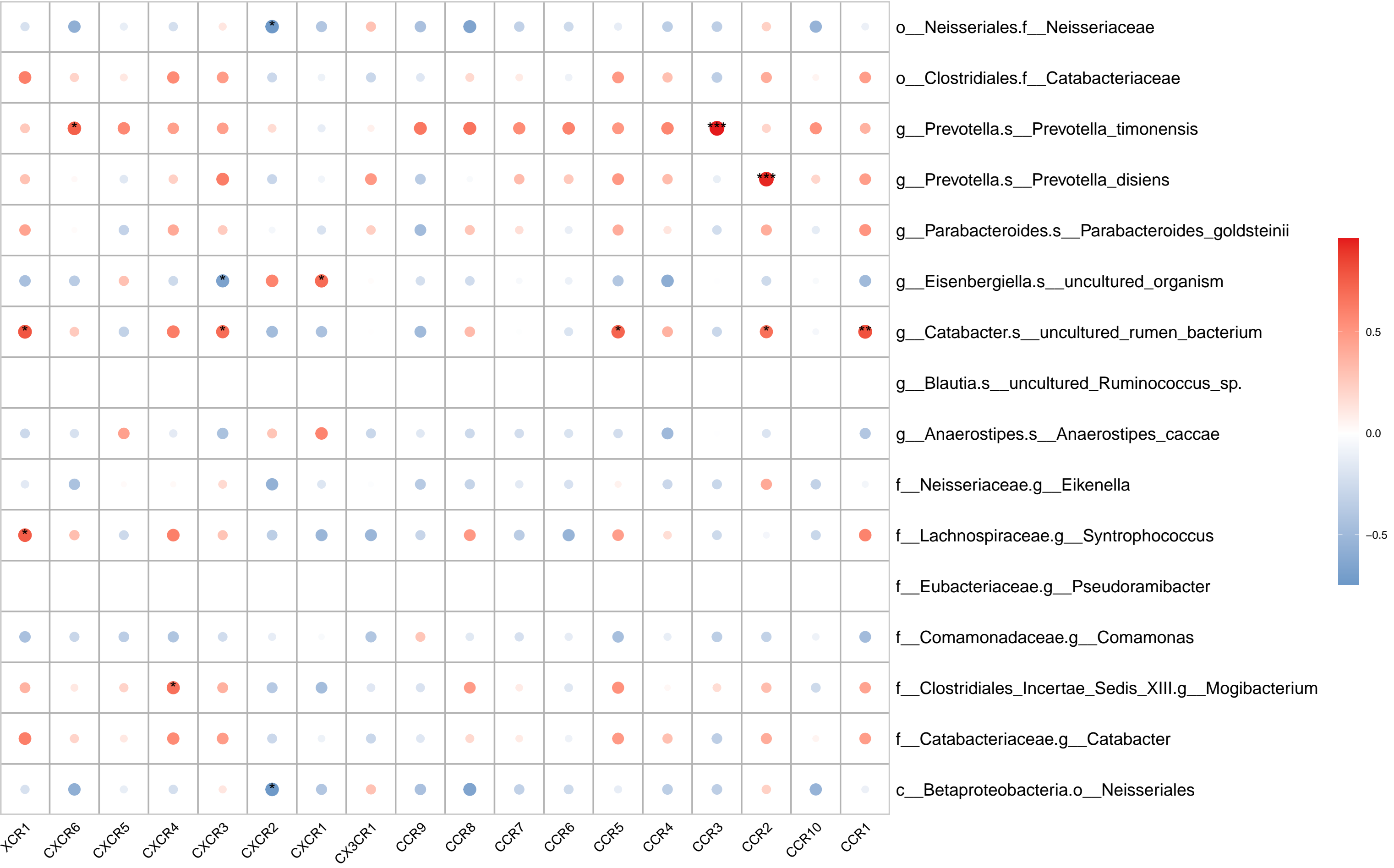

Supplement: Supplementary file 3 — Additional file 3: Figure S3. Heat map of correlation between dominant bacteria and chemokine receptors in the Normal weight group. [file 12967_2024_4903_MOESM3_ESM.pdf]

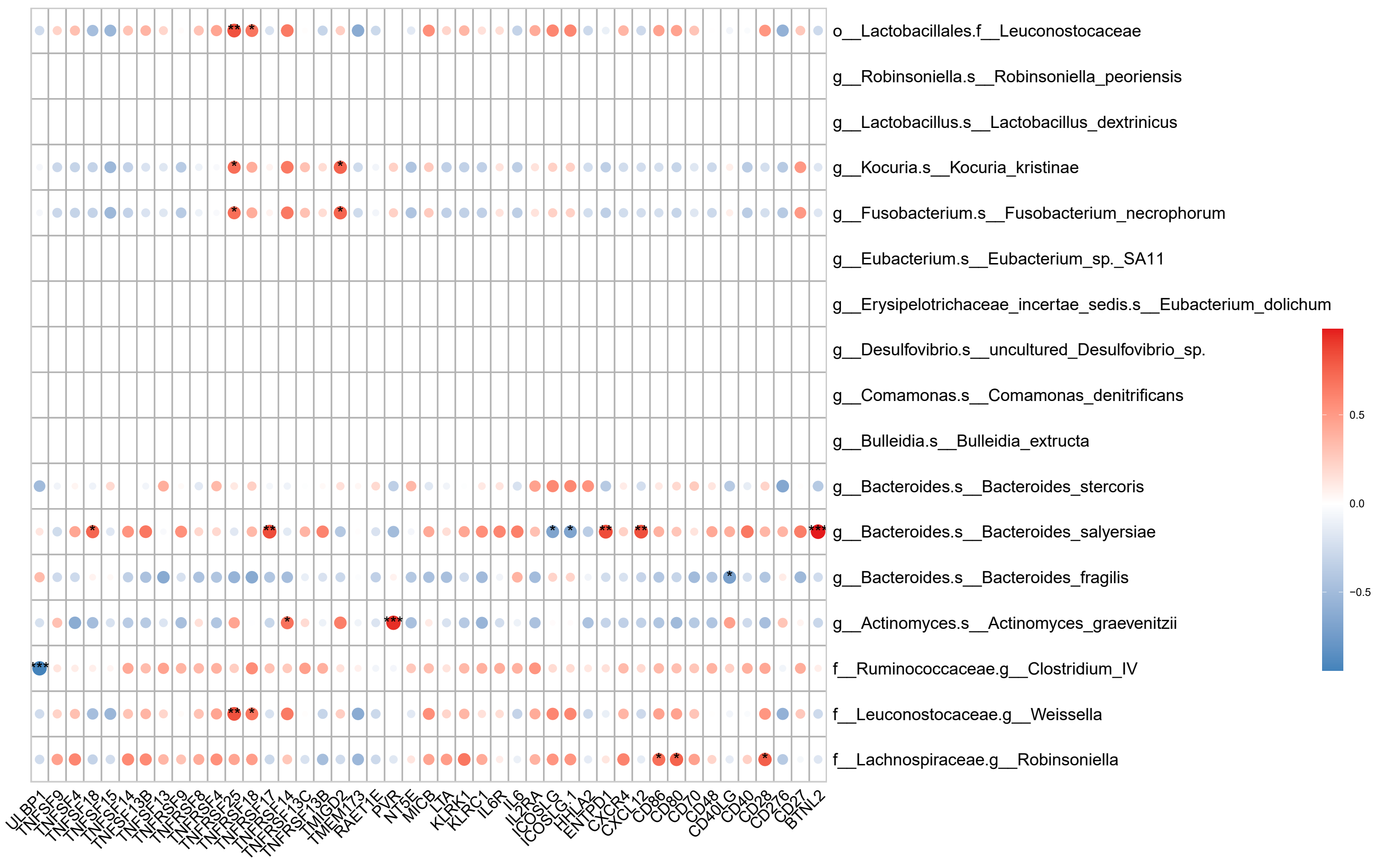

Supplement: Supplementary file 4 — Additional file 4: Figure S4. Heat map of correlation between dominant bacteria and immune activation genes in the Overweight group. [file 12967_2024_4903_MOESM4_ESM.pdf]

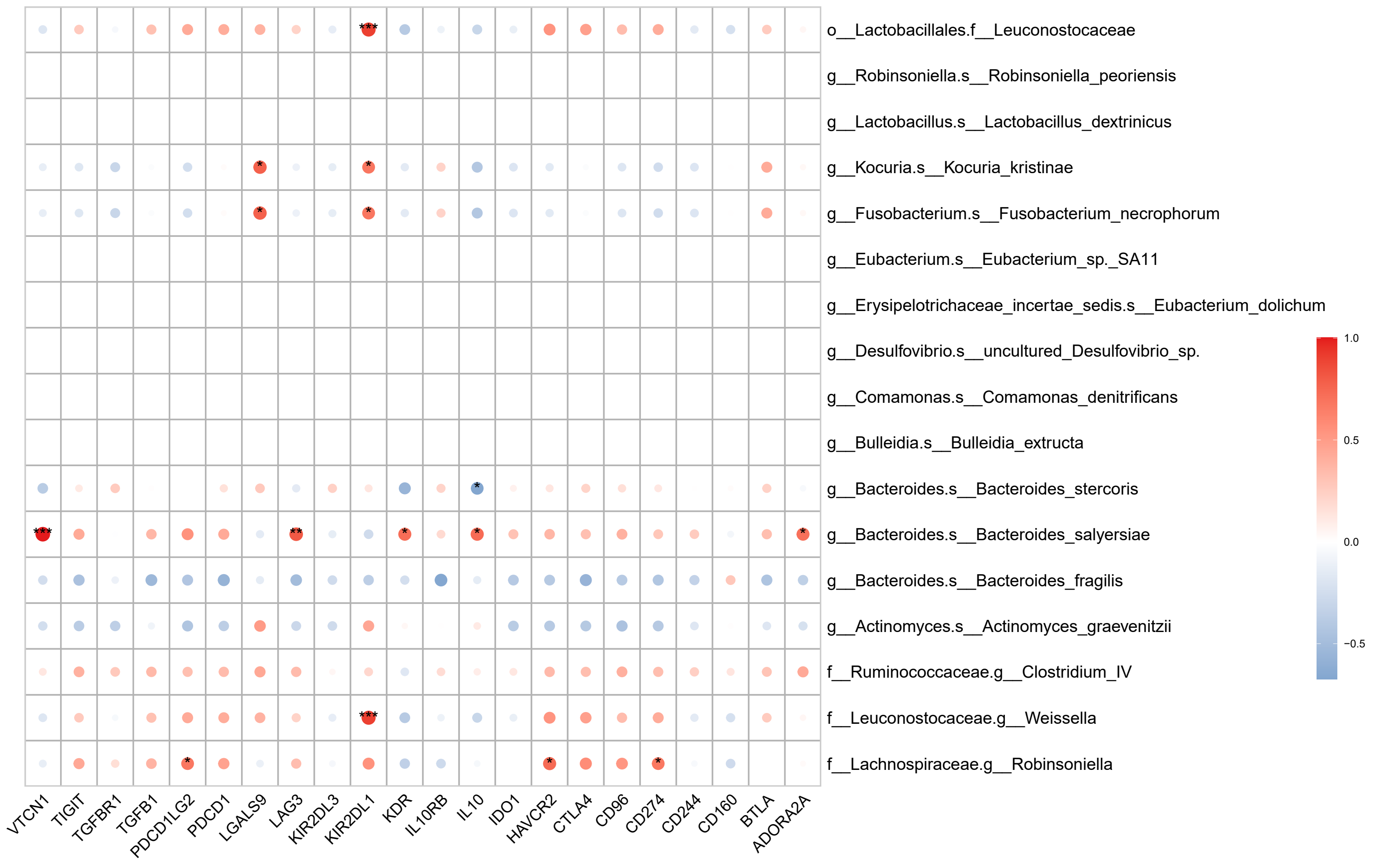

Supplement: Supplementary file 5 — Additional file 5: Figure S5. Heat map of correlation between dominant bacteria and immunosuppressive genes in the Overweight group. [file 12967_2024_4903_MOESM5_ESM.pdf]

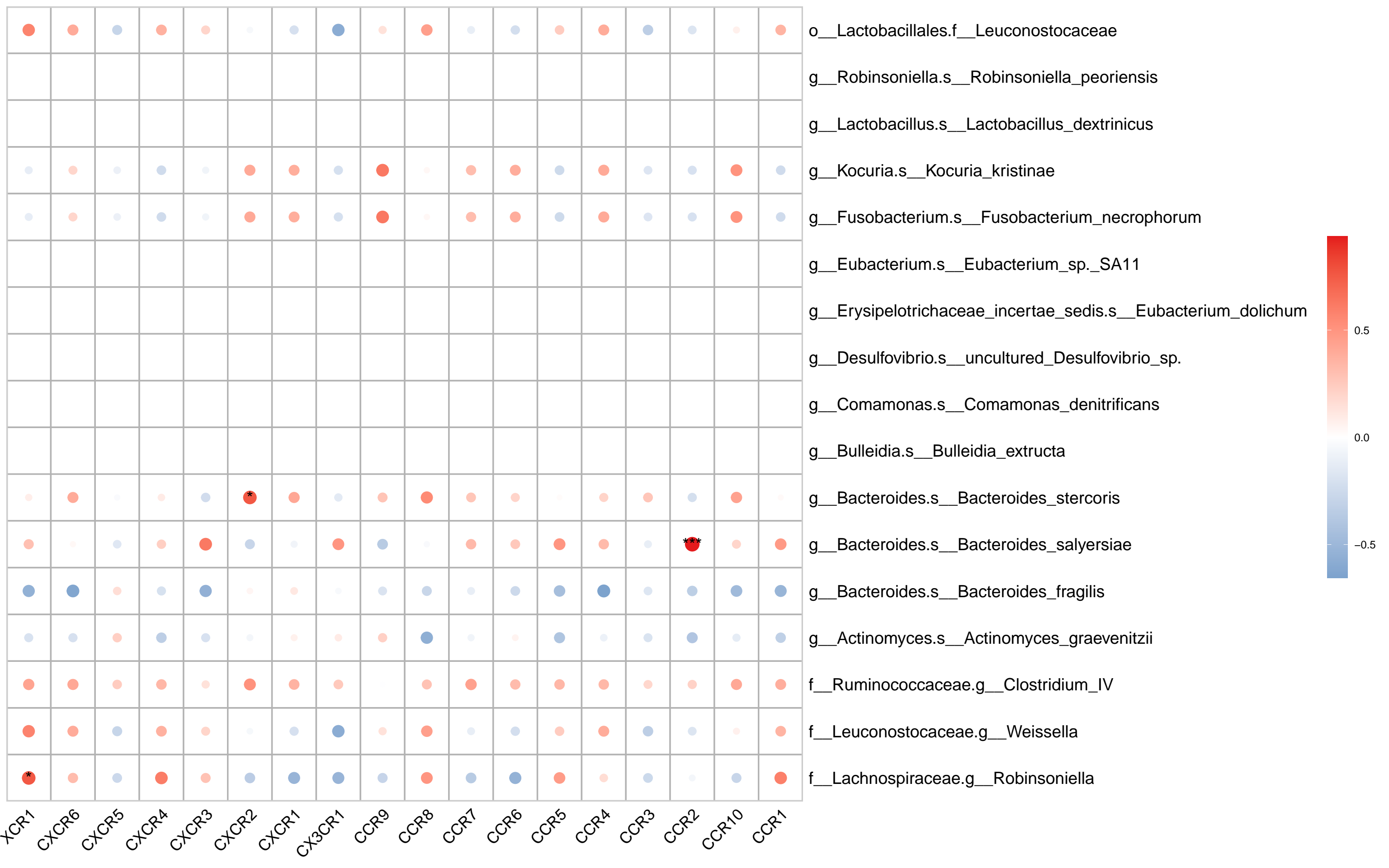

Supplement: Supplementary file 6 — Additional file 6: Figure S6. Heat map of correlation between dominant bacteria and chemokine receptors in the Overweight group. Horizontal coordinate is gene, vertical coordinate is colony, red represents positive correlation, blue represents negative correlation, color depth represents Pearson correlation coefficient size, color from light to dark indicates Pearson correlation coefficient value from small to large. The "*" in the graph represents the size of the p-value: No * for P-value ≥ 0.05, * for 0.01 ≤ P < 0.05, ** for 0.001 ≤ P < 0.01, *** for P < 0.001. [file 12967_2024_4903_MOESM6_ESM.pdf]
