# Supplementary material for: Analysis of differences in intestinal flora associated with different BMI status in colorectal cancer patients
Source: J Transl Med. 2024 Feb 9;22:142. doi: 10.1186/s12967-024-04903-7 (PMC10854193; doi:10.1186/s12967-024-04903-7)
Supplement: Supplementary file 7 — Additional file 7: Table S1. ADONIS test for Bray Distance of intestinal flora in CRC patients in the Overweight and Normal weight groups. [file 12967_2024_4903_MOESM7_ESM.docx]

**Additional file 7：Table S1. ADONIS test for Bray Distance of intestinal flora in CRC patients in the Overweight and Normal weight groups**

|  | **Df** | **Sums Of Sqs** | **Mean Sqs** | **F.Model** | **R2** | **Pr(>F)** |
| --- | --- | --- | --- | --- | --- | --- |
| Group | 1 | 0.37896477 | 0.378964779 | 0.82996959 | 0.004916009 | 0.863 |
| Residuals | 168 | 76.7089342 | 0.456600798 |  | 0.995083991 |  |
| Total | 169 | 77.0878989 |  |  | 1 |  |
