# Supplementary material for: Analysis of differences in intestinal flora associated with different BMI status in colorectal cancer patients
Source: J Transl Med. 2024 Feb 9;22:142. doi: 10.1186/s12967-024-04903-7 (PMC10854193; doi:10.1186/s12967-024-04903-7)
Supplement: Supplementary file 8 — Additional file 8: Table S2. ADONIS test for Jaccard Distance of intestinal flora in CRC patients in the Overweight and Normal weight groups. Group row: between-group statistics; Df: degrees of freedom, between-groups degrees of freedom as number of groups-1, within-groups degrees of freedom as total number of samples—number of groups; Residuals row: statistical information within the group; Total row: intergroup + intragroup statistics; Sums Of Sqs: sum of squares of deviation; Mean Sqs: Mean square, the ratio of the sum of squared deviations to the degrees of freedom, i.e. Sums Of Sqs/Df; F.Model: F test value, i.e. mean square between groups/mean square within groups; R2: the proportion of the sum of squares of deviations between groups and within groups to the sum of squares of total deviations, indicating the degree of explanation for inter-sample differences. The larger R2 indicates the higher degree of explanation for inter-sample differences. Pr(> F): statistically significant P-values derived from replacement tests, Pr < 0.05 was considered statistically significant. [file 12967_2024_4903_MOESM8_ESM.docx]

**Additional file 8：Table S2. ADONIS test for Jaccard Distance of intestinal flora in CRC patients in the Overweight and Normal weight groups**

|  | **Df** | **Sums Of Sqs** | **Mean Sqs** | **F.Model** | **R2** | **Pr(>F)** |
| --- | --- | --- | --- | --- | --- | --- |
| Group | 1 | 0.36903742 | 0.369037426 | 0.861781702 | 0.005103474 | 0.915 |
| Residuals | 168 | 71.9419865 | 0.428226111 |  | 0.994896526 |  |
| Total | 169 | 72.3110239 |  |  | 1 |  |
