# Supplementary material for: Analysis of differences in intestinal flora associated with different BMI status in colorectal cancer patients
Source: J Transl Med. 2024 Feb 9;22:142. doi: 10.1186/s12967-024-04903-7 (PMC10854193; doi:10.1186/s12967-024-04903-7)
Supplement: Supplementary file 9 — Additional file 9: Table S3. Results of LEfSe analysis of intestinal flora between Normal weight and Overweight groups CRC patients. Taxonomy: BMI related intestinal flora information; Group: group with significant abundance of differential species; LDA: effect value of BMI-associated gut flora after log10 treatment; species with LDA scores (log10) greater than 2 and p-values less than 0.05 are shown in Table. [file 12967_2024_4903_MOESM9_ESM.docx]

| **Taxonomy** | **Group** | **LDA (log10)** | **P value** |
| --- | --- | --- | --- |
| f__Clostridiales_Incertae_Sedis_XIII.g__Mogibacterium | Normal weight | 3.104946 | 0.004347 |
| g__Kocuria.s__Kocuria_kristinae | Overweight | 2.475979 | 0.007741 |
| f__Leuconostocaceae.g__Weissella | Overweight | 3.155488 | 0.008809 |
| g__Actinomyces.s__Actinomyces_graevenitzii | Overweight | 2.139205 | 0.008871 |
| f__Lachnospiraceae.g__Robinsoniella | Overweight | 3.076636 | 0.009018 |
| g__Eubacterium.s__Eubacterium_sp__SA11 | Overweight | 2.244333 | 0.009468 |
| f__Eubacteriaceae.g__Pseudoramibacter | Normal weight | 3.593237 | 0.009630 |
| g__Pseudoramibacter.s__Pseudoramibacter_alactolyticus | Normal weight | 3.600380 | 0.009630 |
| o__Lactobacillales.f__Leuconostocaceae | Overweight | 3.166075 | 0.014503 |
| g__Robinsoniella.s__Robinsoniella_peoriensis | Overweight | 3.115730 | 0.015723 |
| g__Erysipelotrichaceae_incertae_sedis.s__Eubacterium_dolichum | Overweight | 2.007575 | 0.019387 |
| g__Haemophilus.s__Haemophilus_sp__paraurethrae | Overweight | 3.922731 | 0.021474 |
| g__Bulleidia.s__Bulleidia_extructa | Overweight | 3.177518 | 0.021474 |
| g__Desulfovibrio.s__uncultured_Desulfovibrio_sp_ | Overweight | 3.154028 | 0.021474 |
| f__Catabacteriaceae.g__Catabacter | Normal weight | 2.735936 | 0.023908 |
| o__Clostridiales.f__Catabacteriaceae | Normal weight | 2.740241 | 0.023908 |
| g__Bacteroides.s__Bacteroides_fragilis | Overweight | 4.955819 | 0.027468 |
| g__Parabacteroides.s__Parabacteroides_goldsteinii | Normal weight | 3.476129 | 0.027479 |
| g__Catabacter.s__uncultured_rumen_bacterium | Normal weight | 2.136667 | 0.027707 |
| g__Eisenbergiella.s__uncultured_organism | Normal weight | 3.159087 | 0.029575 |
| g__Bacteroides.s__Bacteroides_stercoris | Overweight | 5.184168 | 0.033043 |
| f__Comamonadaceae.g__Comamonas | Normal weight | 5.293523 | 0.036163 |
| f__Neisseriaceae.g__Eikenella | Normal weight | 2.761843 | 0.039036 |
| g__Lactobacillus.s__Lactobacillus_dextrinicus | Overweight | 2.795203 | 0.040736 |
| g__Fusobacterium.s__Fusobacterium_necrophorum | Overweight | 3.759421 | 0.040736 |
| g__Prevotella.s__Prevotella_timonensis | Normal weight | 2.203622 | 0.041290 |
| f__Lachnospiraceae.g__Syntrophococcus | Normal weight | 2.397249 | 0.041290 |
| g__Prevotella.s__Prevotella_disiens | Normal weight | 2.127663 | 0.041290 |
| c__Betaproteobacteria.o__Neisseriales | Normal weight | 2.913375 | 0.043730 |
| o__Neisseriales.f__Neisseriaceae | Normal weight | 2.936072 | 0.043730 |
| g__Bacteroides.s__Bacteroides_salyersiae | Overweight | 3.956988 | 0.044843 |
| f__Ruminococcaceae.g__Clostridium_IV | Overweight | 4.305046 | 0.047979 |
| g__Anaerostipes.s__Anaerostipes_caccae | Normal weight | 3.287267 | 0.049328 |

**Additional file 9：Table S3. Results of LEfSe analysis**
