# Supplementary material for: Analysis of differences in intestinal flora associated with different BMI status in colorectal cancer patients
Source: J Transl Med. 2024 Feb 9;22:142. doi: 10.1186/s12967-024-04903-7 (PMC10854193; doi:10.1186/s12967-024-04903-7)
Supplement: Supplementary file 10 — Additional file 10: Table S4. KEGG functional pathways in the intestinal microbiome of CRC patients in the Overweight and Normal weight. KEGG_Pathway: KEGG pathway; Mean In Normal weight: the predicted abundance value of this pathway in each sample in the Normal weight group; Mean In Overweight: the predicted abundance value of this pathway in each sample in the Overweight group. Statistically significant when p-value is less than 0.05. [file 12967_2024_4903_MOESM10_ESM.docx]

**Additional file 10：Table S4. KEGG functional pathways in the intestinal microbiome of CRC patients in the Overweight and Normal weight groups**

| **KEGG_pathway** | **Mean In Normal weight** | **Mean In Overweight** | **P value** |
| --- | --- | --- | --- |
| ko00514:Other types of O-glycan biosynthesis | 249864.52 | 258683.8288 | 0.007631982 |
| ko05110:Vibrio cholerae infection | 2455075.051 | 43192.03754 | 0.022466885 |
| ko00830:Retinol metabolism | 56964362.32 | 17417695.96 | 0.060915097 |
| ko00523:Polyketide sugar unit biosynthesis | 175171069.8 | 36998504.22 | 0.066304955 |
| ko04080:Neuroactive ligand-receptor interaction | 4.650885185 | 0 | 0.12783753 |
| ko00591:Linoleic acid metabolism | 170260241.8 | 76527654.45 | 0.127979886 |
| ko00380:Tryptophan metabolism | 236467978.4 | 176400573.6 | 0.13772228 |
| ko04110:Cell cycle | 2763.28935 | 604.9106694 | 0.207597562 |
| ko00364:Fluorobenzoate degradation | 31871044.96 | 7650445.418 | 0.233895773 |
| ko05143:African trypanosomiasis | 7631027.604 | 3511159.627 | 0.247132401 |
| ko04614:Renin-angiotensin system | 4235.303158 | 2052.071666 | 0.249341711 |
| ko03450:Non-homologous end-joining | 20809714.77 | 16643003.78 | 0.257869399 |
| ko00281:Geraniol degradation | 228042962.9 | 61931282.4 | 0.272140454 |
| ko00903:Limonene and pinene degradation | 57687742.55 | 30638749.75 | 0.28387895 |
| ko04512:ECM-receptor interaction | 597.337963 | 0 | 0.286416657 |
| ko03050:Proteasome | 2894164.083 | 1124028.972 | 0.332258086 |
| ko04974:Protein digestion and absorption | 55175737.75 | 112809877.7 | 0.346992036 |
| ko02040:Flagellar assembly | 537307710.8 | 354253367 | 0.351994376 |
| ko00906:Carotenoid biosynthesis | 20512878.56 | 10777601.35 | 0.368996492 |
| ko00930:Caprolactam degradation | 72423308.64 | 23444941.82 | 0.386500288 |
| ko00623:Toluene degradation | 102467028.2 | 221396457.5 | 0.392519997 |
| ko00965:Betalain biosynthesis | 12511.57245 | 28204.74637 | 0.410200154 |
| ko00785:Lipoic acid metabolism | 1274114626 | 1768526261 | 0.417395996 |
| ko00196:Photosynthesis - antenna proteins | 24168.64021 | 162242.4889 | 0.42204685 |
| ko00524:Butirosin and neomycin biosynthesis | 73893155.1 | 261463831.8 | 0.42494586 |
| ko00626:Naphthalene degradation | 63978198.82 | 155836949.9 | 0.428110516 |
| ko00130:Ubiquinone and other terpenoid-quinone biosynthesis | 492353466 | 596045840.3 | 0.441975173 |
| ko01057:Biosynthesis of type II polyketide products | 71570.60648 | 17827.70565 | 0.450648651 |
| ko00331:Clavulanic acid biosynthesis | 16906.85957 | 0 | 0.455995999 |
| ko00563:Glycosylphosphatidylinositol(GPI)-anchor biosynthesis | 5.131053704 | 0 | 0.455995999 |
| ko00909:Sesquiterpenoid biosynthesis | 25746.82731 | 0 | 0.455995999 |
| ko01056:Biosynthesis of type II polyketide backbone | 73692.54475 | 0 | 0.455995999 |
| ko04520:Adherens junction | 0.761419444 | 0 | 0.455995999 |
| ko05010:Alzheimer's disease | 5666.805621 | 0 | 0.455995999 |
| ko00531:Glycosaminoglycan degradation | 956572162.9 | 1135156249 | 0.469348365 |
| ko05111:Vibrio cholerae pathogenic cycle | 168628476.9 | 210247337.3 | 0.469348365 |
| ko00980:Metabolism of xenobiotics by cytochrome P450 | 20853168.24 | 3289645.867 | 0.471442966 |
| ko00621:Dioxin degradation | 242173875 | 213754861.7 | 0.481360644 |
| ko05322:Systemic lupus erythematosus | 86222.29932 | 14886.31475 | 0.483127464 |
| ko01053:Biosynthesis of siderophore group nonribosomal peptides | 85846382.42 | 79426861.09 | 0.483378835 |
| ko04146:Peroxisome | 253494935.8 | 292873124.8 | 0.505879057 |
| ko04210:Apoptosis | 43558974.92 | 88589316.34 | 0.507951519 |
| ko03008:Ribosome biogenesis in eukaryotes | 62784158.18 | 72209103.15 | 0.510028427 |
| ko00072:Synthesis and degradation of ketone bodies | 468483602 | 252708916.2 | 0.522582557 |
| ko00195:Photosynthesis | 81063547.72 | 124414256.4 | 0.524211395 |
| ko04020:Calcium signaling pathway | 4951.962244 | 65126.02845 | 0.526949788 |
| ko00071:Fatty acid metabolism | 429425751.5 | 432139739 | 0.528918628 |
| ko00540:Lipopolysaccharide biosynthesis | 885933445 | 1109172502 | 0.539564932 |
| ko00410:beta-Alanine metabolism | 398875730.4 | 608810777.5 | 0.541326439 |
| ko01040:Biosynthesis of unsaturated fatty acids | 420969266.5 | 424420402.4 | 0.548158554 |
| ko02030:Bacterial chemotaxis | 979503964.3 | 710931349.5 | 0.550317478 |
| ko00750:Vitamin B6 metabolism | 1245922428 | 1616870793 | 0.554647846 |
| ko04962:Vasopressin-regulated water reabsorption | 14735.22527 | 4728.507023 | 0.555881949 |
| ko00760:Nicotinate and nicotinamide metabolism | 1217437568 | 1610578747 | 0.556819262 |
| ko00430:Taurine and hypotaurine metabolism | 757921480.8 | 890354191.6 | 0.561174481 |
| ko00020:Citrate cycle (TCA cycle) | 1195083923 | 1492638976 | 0.567738053 |
| ko00640:Propanoate metabolism | 765692732.6 | 831987769.5 | 0.569934047 |
| ko00720:Carbon fixation pathways in prokaryotes | 1286896733 | 1629758415 | 0.569934047 |
| ko00260:Glycine, serine and threonine metabolism | 1214900646 | 1337975340 | 0.572134084 |
| ko00630:Glyoxylate and dicarboxylate metabolism | 786069078.3 | 807498993 | 0.572134084 |
| ko03070:Bacterial secretion system | 860726948.2 | 982967372.5 | 0.572134084 |
| ko00564:Glycerophospholipid metabolism | 657234800.3 | 719642779.7 | 0.574338149 |
| ko00770:Pantothenate and CoA biosynthesis | 1964270974 | 2264765228 | 0.576546224 |
| ko02020:Two-component system | 397028401.4 | 371063623.7 | 0.576546224 |
| ko05012:Parkinson's disease | 6136152.976 | 1189811.11 | 0.577232497 |
| ko00642:Ethylbenzene degradation | 43696734.11 | 58440970.63 | 0.579538282 |
| ko00450:Selenocompound metabolism | 1238568182 | 1340552124 | 0.580974346 |
| ko00710:Carbon fixation in photosynthetic organisms | 1728839735 | 2082482745 | 0.580974346 |
| ko03410:Base excision repair | 1025653565 | 1172181525 | 0.580974346 |
| ko00900:Terpenoid backbone biosynthesis | 1445397931 | 1756801392 | 0.58319436 |
| ko00280:Valine, leucine and isoleucine degradation | 504145259.3 | 541144131.3 | 0.585418321 |
| ko00350:Tyrosine metabolism | 304864760.7 | 271598260 | 0.585418321 |
| ko00362:Benzoate degradation | 201696097.8 | 188825216 | 0.585418321 |
| ko00550:Peptidoglycan biosynthesis | 2064656712 | 2472047512 | 0.585418321 |
| ko00970:Aminoacyl-tRNA biosynthesis | 1824677928 | 2177664209 | 0.585418321 |
| ko03018:RNA degradation | 704145409.3 | 854006183.3 | 0.585418321 |
| ko04626:Plant-pathogen interaction | 210075992.1 | 229362348 | 0.585418321 |
| ko00190:Oxidative phosphorylation | 539807339.9 | 579664440.2 | 0.587646212 |
| ko03440:Homologous recombination | 1667915498 | 2004234756 | 0.587646212 |
| ko04910:Insulin signaling pathway | 104438145.6 | 119720228.6 | 0.587646212 |
| ko00240:Pyrimidine metabolism | 1293642859 | 1585104826 | 0.592113722 |
| ko00361:Chlorocyclohexane and chlorobenzene degradation | 90097357.02 | 66903145 | 0.592113722 |
| ko00650:Butanoate metabolism | 776369175.6 | 876870814.3 | 0.592113722 |
| ko00791:Atrazine degradation | 121553551.9 | 69492824.25 | 0.594340698 |
| ko00730:Thiamine metabolism | 1836165267 | 2148155075 | 0.594353307 |
| ko04141:Protein processing in endoplasmic reticulum | 51854905.01 | 70702748.18 | 0.594353307 |
| ko00230:Purine metabolism | 989465750.7 | 1165595902 | 0.596596757 |
| ko00473:D-Alanine metabolism | 1893691374 | 2241446841 | 0.596596757 |
| ko02010:ABC transporters | 777931826.9 | 750210003.2 | 0.596596757 |
| ko00471:D-Glutamine and D-glutamate metabolism | 2254880445 | 2863272134 | 0.598844054 |
| ko00290:Valine, leucine and isoleucine biosynthesis | 2437700649 | 2488543893 | 0.601095181 |
| ko00627:Aminobenzoate degradation | 168266798.9 | 207160466.1 | 0.601095181 |
| ko03040:Spliceosome | 23063.54723 | 83636.73761 | 0.601670816 |
| ko00310:Lysine degradation | 230581767.8 | 252844307.2 | 0.603350122 |
| ko00480:Glutathione metabolism | 541545594.4 | 557828109.7 | 0.603350122 |
| ko03030:DNA replication | 1362646695 | 1648787491 | 0.603350122 |
| ko03060:Protein export | 1606447317 | 1932251645 | 0.603350122 |
| ko03430:Mismatch repair | 1887396179 | 2260148097 | 0.603350122 |
| ko00620:Pyruvate metabolism | 1316574644 | 1419636859 | 0.605608859 |
| ko00910:Nitrogen metabolism | 697441616.1 | 779706951 | 0.607871374 |
| ko05410:Hypertrophic cardiomyopathy (HCM) | 740861.0672 | 1122582.222 | 0.60801748 |
| ko00270:Cysteine and methionine metabolism | 1394837796 | 1478845744 | 0.610137651 |
| ko04122:Sulfur relay system | 1144492967 | 1261416521 | 0.610137651 |
| ko00920:Sulfur metabolism | 910652789.6 | 879250270.9 | 0.612407672 |
| ko00010:Glycolysis / Gluconeogenesis | 1265370314 | 1440246926 | 0.616958875 |
| ko00908:Zeatin biosynthesis | 747095558.5 | 1055977353 | 0.616958875 |
| ko03420:Nucleotide excision repair | 902092443.8 | 1066418260 | 0.619240022 |
| ko00790:Folate biosynthesis | 1400428787 | 2026657119 | 0.621524841 |
| ko03010:Ribosome | 1741992329 | 2155641468 | 0.621524841 |
| ko00960:Tropane, piperidine and pyridine alkaloid biosynthesis | 438022580.1 | 412840010.2 | 0.623813314 |
| ko00680:Methane metabolism | 589050386 | 701747882.9 | 0.626105424 |
| ko00400:Phenylalanine, tyrosine and tryptophan biosynthesis | 1317191682 | 1422259713 | 0.628401152 |
| ko03020:RNA polymerase | 1153454081 | 1284400482 | 0.628401152 |
| ko00670:One carbon pool by folate | 1977100637 | 2399759485 | 0.63070048 |
| ko00030:Pentose phosphate pathway | 1806054387 | 2130009102 | 0.633003389 |
| ko00330:Arginine and proline metabolism | 818123824.6 | 866103737.3 | 0.633003389 |
| ko04112:Cell cycle - Caulobacter | 1682022760 | 2085450439 | 0.633003389 |
| ko04113:Meiosis - yeast | 405531.8653 | 123164.8183 | 0.634173265 |
| ko00740:Riboflavin metabolism | 962474305.1 | 1354243800 | 0.637619877 |
| ko00561:Glycerolipid metabolism | 547525166.6 | 539247070.4 | 0.639933418 |
| ko00460:Cyanoamino acid metabolism | 233870176.4 | 190506910.4 | 0.641526816 |
| ko00300:Lysine biosynthesis | 1743435797 | 1986863516 | 0.642250465 |
| ko01051:Biosynthesis of ansamycins | 4575889299 | 5071142635 | 0.644570999 |
| ko01055:Biosynthesis of vancomycin group antibiotics | 2736681601 | 3371001609 | 0.644570999 |
| ko00061:Fatty acid biosynthesis | 1773337340 | 2002341997 | 0.649222453 |
| ko00622:Xylene degradation | 38533357.46 | 41147101.32 | 0.653073905 |
| ko00250:Alanine, aspartate and glutamate metabolism | 1864233870 | 2171324398 | 0.653887625 |
| ko00520:Amino sugar and nucleotide sugar metabolism | 1250574710 | 1507282478 | 0.653887625 |
| ko04310:Wnt signaling pathway | 962.1166852 | 24.43745806 | 0.655110166 |
| ko00120:Primary bile acid biosynthesis | 136022938.2 | 156938741.8 | 0.656225307 |
| ko00121:Secondary bile acid biosynthesis | 1218030062 | 1409766309 | 0.656225307 |
| ko00040:Pentose and glucuronate interconversions | 754315009.7 | 835689322.8 | 0.660910765 |
| ko00660:C5-Branched dibasic acid metabolism | 1955653936 | 1968757475 | 0.665609549 |
| ko04970:Salivary secretion | 99531.16512 | 149271.9398 | 0.667233723 |
| ko02060:Phosphotransferase system (PTS) | 432089794.2 | 450218304.1 | 0.667963888 |
| ko05100:Bacterial invasion of epithelial cells | 9846795.196 | 5597778.412 | 0.667963888 |
| ko00500:Starch and sucrose metabolism | 1235501871 | 1435301299 | 0.672682361 |
| ko04621:NOD-like receptor signaling pathway | 92649247.3 | 120595033.7 | 0.675046454 |
| ko05120:Epithelial cell signaling in Helicobacter pylori infection | 185872385.9 | 221267069.3 | 0.677413758 |
| ko00053:Ascorbate and aldarate metabolism | 309691945.5 | 311074116.6 | 0.684534729 |
| ko00643:Styrene degradation | 54098763.36 | 39825410.77 | 0.685854832 |
| ko00340:Histidine metabolism | 1338581052 | 1519668661 | 0.689297719 |
| ko00440:Phosphonate and phosphinate metabolism | 157982962.8 | 168258145.8 | 0.689297719 |
| ko03015:mRNA surveillance pathway | 46775.8858 | 48782.45699 | 0.692970487 |
| ko00051:Fructose and mannose metabolism | 1180891308 | 1380152978 | 0.694073054 |
| ko00511:Other glycan degradation | 2158341174 | 2597814016 | 0.694073054 |
| ko00941:Flavonoid biosynthesis | 8539201.888 | 10636111.23 | 0.694073054 |
| ko05145:Toxoplasmosis | 48455.65178 | 55727.29811 | 0.699200321 |
| ko00360:Phenylalanine metabolism | 341859985.5 | 307031505 | 0.701258834 |
| ko00521:Streptomycin biosynthesis | 1990627798 | 2509314793 | 0.701258834 |
| ko00780:Biotin metabolism | 1762024086 | 2524317516 | 0.720550459 |
| ko00860:Porphyrin and chlorophyll metabolism | 750188469.7 | 1019074502 | 0.72297477 |
| ko00140:Steroid hormone biosynthesis | 65520764.17 | 95222143.12 | 0.730264313 |
| ko05146:Amoebiasis | 18677785.81 | 38968026.88 | 0.737578306 |
| ko00562:Inositol phosphate metabolism | 236944006 | 256270695.8 | 0.742467584 |
| ko00052:Galactose metabolism | 1333791578 | 1492247274 | 0.744916138 |
| ko03013:RNA transport | 58327225.86 | 80854323.85 | 0.749820966 |
| ko00600:Sphingolipid metabolism | 610071761.3 | 704296596.5 | 0.759660847 |
| ko00100:Steroid biosynthesis | 26490741.93 | 17814119.03 | 0.764595388 |
| ko00983:Drug metabolism - other enzymes | 172882266.8 | 464963716.7 | 0.770506475 |
| ko00311:Penicillin and cephalosporin biosynthesis | 37882502.87 | 33286892.46 | 0.776973714 |
| ko04075:Plant hormone signal transduction | 341222.533 | 161902.4177 | 0.794279035 |
| ko00472:D-Arginine and D-ornithine metabolism | 98369016.41 | 81019384.96 | 0.794398067 |
| ko05130:Pathogenic Escherichia coli infection | 28384.31238 | 10005.74787 | 0.800784288 |
| ko00625:Chloroalkane and chloroalkene degradation | 219951949.7 | 144470873.6 | 0.803621783 |
| ko00633:Nitrotoluene degradation | 358804496.6 | 316752105.9 | 0.84725643 |
| ko00510:N-Glycan biosynthesis | 56684485.58 | 84772443.6 | 0.865042386 |
| ko04144:Endocytosis | 505538.105 | 483046.2482 | 0.882895554 |
| ko00513:Various types of N-glycan biosynthesis | 43873.34537 | 409.3703226 | 0.90396431 |
| ko05142:Chagas disease (American trypanosomiasis) | 133822.8491 | 15458.42183 | 0.90396431 |
| ko00312:beta-Lactam resistance | 271410810.3 | 269192143.6 | 0.913638722 |
| ko05144:Malaria | 13669.75239 | 2403.187305 | 0.915217163 |
| ko00590:Arachidonic acid metabolism | 41567.60724 | 254406.6653 | 0.926487034 |
| ko00601:Glycosphingolipid biosynthesis - lacto and neolacto series | 253038.3876 | 1595884.159 | 0.946623434 |
| ko00363:Bisphenol degradation | 88489044.83 | 17692947.64 | 0.95722599 |
| ko00943:Isoflavonoid biosynthesis | 14650.78825 | 17270.56452 | 0.973900288 |
| ko04142:Lysosome | 20893312.59 | 384268.9429 | 0.974033484 |
| ko00624:Polycyclic aromatic hydrocarbon degradation | 4547867.441 | 488736.4869 | 0.977334372 |
| ko05150:Staphylococcus aureus infection | 35554671.33 | 38348206.99 | 0.990959683 |
