# Supplementary material for: Analysis of differences in intestinal flora associated with different BMI status in colorectal cancer patients
Source: J Transl Med. 2024 Feb 9;22:142. doi: 10.1186/s12967-024-04903-7 (PMC10854193; doi:10.1186/s12967-024-04903-7)
Supplement: Supplementary file 11 — Additional file 11: Table S5. List of differential KEGG pathways of CRC patients stratified by BMI condition. KEGG pathway: enriched KEGG pathway. logFC: FC represents the folding change, that is, the ratio of the expression of the Overweight group and the Normal weight group. The logarithm is taken as the base of 2. Statistically significant when p-value is less than 0.05. [file 12967_2024_4903_MOESM11_ESM.docx]

**Additional file 11：Table S5. List of differential KEGG pathways of CRC patients stratified by BMI condition**

| **KEGG Pathways** | **logFC** | **P.Value** |
| --- | --- | --- |
| KEGG_TYPE_II_DIABETES_MELLITUS | -0.03833 | 0.01405 |
| KEGG_PROTEIN_EXPORT | 0.08208 | 0.01982 |
