# Supplementary material for: Analysis of differences in intestinal flora associated with different BMI status in colorectal cancer patients
Source: J Transl Med. 2024 Feb 9;22:142. doi: 10.1186/s12967-024-04903-7 (PMC10854193; doi:10.1186/s12967-024-04903-7)
Supplement: Supplementary file 12 — Additional file 12: Table S6. List of differential GO items of CRC patients stratified by BMI condition. GO items: Enriched GO entries. LogFC: FC represents the folding change, that is, the ratio of the expression of the Overweight group and the Normal weight group. The logarithm is taken as the base of 2. Statistically significant when p-value is less than 0.05. [file 12967_2024_4903_MOESM12_ESM.docx]

**Additional file 12：Table S6. List of differential GO items of CRC patients stratified by BMI condition**

| **GO items** | **LogFC** | **P.Value** |
| --- | --- | --- |
| GOMF_CYCLIN_DEPENDENT_PROTEIN_SERINE_THREONINE_KINASE_INHIBITOR_ACTIVITY | -0.118133675 | 0.001712877 |
| GOCC_DENDRITIC_SPINE_MEMBRANE | -0.064228479 | 0.00221485 |
| GOBP_PROTEIN_DENEDDYLATION | 0.057526758 | 0.002274157 |
| GOBP_NEGATIVE_REGULATION_OF_CYCLIN_DEPENDENT_PROTEIN_SERINE_THREONINE_KINASE_ACTIVITY | -0.058362761 | 0.002334494 |
| GOMF_RNA_POLYMERASE_II_GENERAL_TRANSCRIPTION_INITIATION_FACTOR_BINDING | 0.045752758 | 0.002674185 |
| GOMF_SULFUR_AMINO_ACID_TRANSMEMBRANE_TRANSPORTER_ACTIVITY | -0.051734419 | 0.004091487 |
| GOBP_PURINE_NUCLEOTIDE_SALVAGE | 0.067690684 | 0.004794164 |
| GOBP_COPPER_ION_TRANSPORT | 0.056178564 | 0.005289813 |
| GOMF_POTASSIUM_ION_BINDING | -0.053058165 | 0.005417039 |
| GOBP_HISTAMINE_TRANSPORT | 0.055554134 | 0.005647357 |
| GOBP_PURINE_CONTAINING_COMPOUND_SALVAGE | 0.061549779 | 0.005655526 |
| GOBP_PROTEIN_NEDDYLATION | 0.046409593 | 0.006111195 |
| GOBP_CYTIDINE_METABOLIC_PROCESS | 0.073146276 | 0.006378432 |
| GOMF_CYTIDINE_DEAMINASE_ACTIVITY | 0.073146276 | 0.006378432 |
| GOBP_REPRODUCTIVE_BEHAVIOR | -0.044435135 | 0.006498108 |
| GOMF_PROTEIN_SERINE_THREONINE_KINASE_INHIBITOR_ACTIVITY | -0.065243747 | 0.006728587 |
| GOBP_RIBONUCLEOSIDE_METABOLIC_PROCESS | 0.058384458 | 0.007689216 |
| GOBP_REGULATION_OF_UNSATURATED_FATTY_ACID_BIOSYNTHETIC_PROCESS | 0.085309182 | 0.008022502 |
| GOBP_MATING_BEHAVIOR | -0.051117694 | 0.008635343 |
| GOMF_TFIID_CLASS_TRANSCRIPTION_FACTOR_COMPLEX_BINDING | 0.038813923 | 0.009247975 |
| GOBP_REGULATION_OF_PROTEIN_NEDDYLATION | 0.041633353 | 0.009700465 |
| GOBP_POSITIVE_REGULATION_OF_FATTY_ACID_BIOSYNTHETIC_PROCESS | 0.047825872 | 0.010586095 |
| GOBP_POSITIVE_REGULATION_OF_MYOBLAST_PROLIFERATION | 0.045888017 | 0.010653803 |
| GOMF_K48_LINKED_POLYUBIQUITIN_MODIFICATION_DEPENDENT_PROTEIN_BINDING | -0.035461679 | 0.010730076 |
| GOBP_PURINE_NUCLEOSIDE_BIOSYNTHETIC_PROCESS | 0.080416104 | 0.011391752 |
| GOBP_ENDOPLASMIC_RETICULUM_MEMBRANE_ORGANIZATION | 0.054134737 | 0.012344508 |
| GOBP_INOSITOL_METABOLIC_PROCESS | -0.061449525 | 0.01265649 |
| GOBP_AUTOPHAGY_OF_NUCLEUS | 0.043629785 | 0.013178501 |
| GOBP_SIGNAL_PEPTIDE_PROCESSING | 0.066905859 | 0.013348869 |
| GOBP_CELLULAR_POLYSACCHARIDE_CATABOLIC_PROCESS | 0.045015743 | 0.013552985 |
| GOBP_HISTAMINE_PRODUCTION_INVOLVED_IN_INFLAMMATORY_RESPONSE | 0.075252832 | 0.014640445 |
| GOBP_POSITIVE_REGULATION_OF_ERAD_PATHWAY | 0.041927679 | 0.015986825 |
| GOBP_AGGREPHAGY | 0.052576997 | 0.016672913 |
| GOCC_GOLGI_TRANS_CISTERNA | 0.051239882 | 0.016852884 |
| GOBP_PROTEIN_TARGETING_TO_MEMBRANE | 0.0286716 | 0.01757081 |
| GOBP_GROWTH_HORMONE_SECRETION | 0.039635787 | 0.017757871 |
| GOBP_COPULATION | -0.047163271 | 0.018146283 |
| GOBP_NUCLEAR_BODY_ORGANIZATION | -0.037661124 | 0.01852512 |
| GOBP_HISTAMINE_SECRETION | 0.069846476 | 0.018665403 |
| GOBP_POSITIVE_REGULATION_OF_VASCULAR_PERMEABILITY | -0.03849901 | 0.01895403 |
| GOBP_POSITIVE_REGULATION_OF_SMOOTH_MUSCLE_CELL_APOPTOTIC_PROCESS | -0.037548462 | 0.019079551 |
| GOBP_CELLULAR_RESPONSE_TO_NITROGEN_STARVATION | 0.045417832 | 0.019236434 |
| GOBP_POSITIVE_REGULATION_OF_PROTEIN_TARGETING_TO_MEMBRANE | 0.03925287 | 0.019442933 |
| GOMF_ATPASE_ACTIVATOR_ACTIVITY | 0.034548722 | 0.019473049 |
| GOBP_CELLULAR_RESPONSE_TO_OXYGEN_RADICAL | 0.039955653 | 0.019609286 |
| GOMF_GLUTAMATE_RECEPTOR_ACTIVITY | -0.03982311 | 0.019829736 |
| GOBP_POLYSACCHARIDE_CATABOLIC_PROCESS | 0.041597649 | 0.020455323 |
| GOBP_NEUROTROPHIN_TRK_RECEPTOR_SIGNALING_PATHWAY | -0.031820197 | 0.020682356 |
| GOBP_POSITIVE_REGULATION_OF_PEPTIDYL_THREONINE_PHOSPHORYLATION | 0.039718686 | 0.021140416 |
| GOBP_REGULATION_OF_MYOBLAST_PROLIFERATION | 0.038959048 | 0.021603979 |
| GOBP_RESPONSE_TO_MERCURY_ION | 0.041962852 | 0.021615073 |
| GOBP_REGULATION_OF_TRANSCRIPTION_FROM_RNA_POLYMERASE_II_PROMOTER_IN_RESPONSE_TO_HYPOXIA | 0.041191815 | 0.022207362 |
| GOBP_CHAPERONE_MEDIATED_PROTEIN_COMPLEX_ASSEMBLY | 0.032176404 | 0.022370049 |
| GOBP_LUTEINIZING_HORMONE_SECRETION | 0.092865395 | 0.023257255 |
| GOBP_GRANULOCYTE_MACROPHAGE_COLONY_STIMULATING_FACTOR_PRODUCTION | 0.057072423 | 0.023401179 |
| GOBP_REGULATION_OF_MAMMARY_GLAND_EPITHELIAL_CELL_PROLIFERATION | -0.040280712 | 0.024135727 |
| GOBP_POSITIVE_REGULATION_OF_GRANULOCYTE_MACROPHAGE_COLONY_STIMULATING_FACTOR_PRODUCTION | 0.058561391 | 0.025002613 |
| GOBP_NEGATIVE_REGULATION_OF_ADENYLATE_CYCLASE_ACTIVATING_G_PROTEIN_COUPLED_RECEPTOR_SIGNALING_PATHWAY | 0.056463428 | 0.025338995 |
| GOMF_DIPEPTIDASE_ACTIVITY | -0.053052943 | 0.02584669 |
| GOBP_MUSCLE_ATROPHY | -0.034266233 | 0.025987456 |
| GOBP_NOREPINEPHRINE_SECRETION | -0.051488075 | 0.026082719 |
| GOBP_INSEMINATION | -0.044902031 | 0.026148386 |
| GOMF_BETA_TUBULIN_BINDING | 0.029160352 | 0.027533322 |
| GOBP_REGULATION_OF_CYCLIN_DEPENDENT_PROTEIN_KINASE_ACTIVITY | -0.031828337 | 0.027749755 |
| GOBP_PURINE_NUCLEOSIDE_MONOPHOSPHATE_BIOSYNTHETIC_PROCESS | 0.04042009 | 0.028018519 |
| GOBP_MAINTENANCE_OF_SISTER_CHROMATID_COHESION | 0.064356389 | 0.02872161 |
| GOBP_RIBONUCLEOSIDE_CATABOLIC_PROCESS | 0.043989755 | 0.028864832 |
| GOBP_G1_TO_G0_TRANSITION | -0.062525764 | 0.029459862 |
| GOBP_POSITIVE_REGULATION_OF_CELL_MIGRATION_INVOLVED_IN_SPROUTING_ANGIOGENESIS | 0.072156358 | 0.030427806 |
| GOMF_GDP_BINDING | 0.035537981 | 0.031150424 |
| GOBP_NEGATIVE_REGULATION_OF_RECEPTOR_BINDING | -0.053448954 | 0.031732754 |
| GOBP_POSITIVE_REGULATION_OF_GLUTAMATE_SECRETION | 0.096924855 | 0.031752468 |
| GOMF_SECONDARY_ACTIVE_MONOCARBOXYLATE_TRANSMEMBRANE_TRANSPORTER_ACTIVITY | 0.034125691 | 0.031785404 |
| GOBP_UREA_TRANSPORT | -0.049554259 | 0.032465536 |
| GOMF_ALPHA_TUBULIN_BINDING | 0.03390143 | 0.032626934 |
| GOBP_FC_EPSILON_RECEPTOR_SIGNALING_PATHWAY | 0.049536004 | 0.033228866 |
| GOMF_HISTONE_METHYLTRANSFERASE_ACTIVITY_H3_K4_SPECIFIC | -0.035118582 | 0.033262673 |
| GOBP_GROOMING_BEHAVIOR | 0.047569518 | 0.033313633 |
| GOBP_PYRIMIDINE_NUCLEOSIDE_TRIPHOSPHATE_BIOSYNTHETIC_PROCESS | -0.037938017 | 0.033593407 |
| GOMF_NEUREXIN_FAMILY_PROTEIN_BINDING | 0.037558389 | 0.033638129 |
| GOBP_CYTIDINE_TO_URIDINE_EDITING | 0.059419249 | 0.033966165 |
| GOBP_REGULATION_OF_HEMATOPOIETIC_STEM_CELL_DIFFERENTIATION | 0.042108512 | 0.034138587 |
| GOBP_GONADOTROPIN_SECRETION | 0.086915726 | 0.034319934 |
| GOBP_UTERINE_SMOOTH_MUSCLE_CONTRACTION | -0.078322713 | 0.034400324 |
| GOBP_GOLGI_LUMEN_ACIDIFICATION | 0.045785713 | 0.034640827 |
| GOBP_PURINE_RIBONUCLEOSIDE_METABOLIC_PROCESS | 0.058849708 | 0.034786179 |
| GOBP_PYRIMIDINE_NUCLEOSIDE_CATABOLIC_PROCESS | 0.059260493 | 0.034880155 |
| GOBP_NEGATIVE_REGULATION_OF_MIRNA_METABOLIC_PROCESS | -0.030798631 | 0.035570174 |
| GOMF_LEUCINE_ZIPPER_DOMAIN_BINDING | -0.04492517 | 0.036032677 |
| GOMF_HISTONE_METHYLTRANSFERASE_BINDING | -0.029489596 | 0.03665096 |
| GOBP_POSITIVE_REGULATION_OF_STEM_CELL_DIFFERENTIATION | 0.037924992 | 0.036796011 |
| GOMF_ACYLGLYCEROL_LIPASE_ACTIVITY | -0.073720403 | 0.037508495 |
| GOMF_SUMO_LIGASE_ACTIVITY | 0.035136474 | 0.03755859 |
| GOMF_QUATERNARY_AMMONIUM_GROUP_TRANSMEMBRANE_TRANSPORTER_ACTIVITY | -0.081143594 | 0.037573828 |
| GOBP_NEGATIVE_REGULATION_OF_CIRCADIAN_RHYTHM | -0.03514267 | 0.037874489 |
| GOMF_CYCLIC_NUCLEOTIDE_GATED_ION_CHANNEL_ACTIVITY | 0.051247608 | 0.039020306 |
| GOBP_LEUKOCYTE_AGGREGATION | 0.077597167 | 0.039203447 |
| GOMF_DEAMINASE_ACTIVITY | 0.037085166 | 0.039641745 |
| GOCC_GAP_JUNCTION | 0.065935629 | 0.039844715 |
| GOBP_REGULATION_OF_POSTSYNAPTIC_DENSITY_ASSEMBLY | -0.056298832 | 0.040457893 |
| GOBP_OUTER_MITOCHONDRIAL_MEMBRANE_ORGANIZATION | 0.052392683 | 0.040805118 |
| GOBP_REGULATION_OF_CYTOPLASMIC_TRANSLATION | 0.032163732 | 0.041092812 |
| GOMF_SOLUTE_PROTON_SYMPORTER_ACTIVITY | -0.028240111 | 0.041167228 |
| GOBP_EMBRYONIC_PLACENTA_MORPHOGENESIS | 0.040497868 | 0.041829022 |
| GOBP_POSITIVE_REGULATION_OF_SMOOTH_MUSCLE_CELL_DIFFERENTIATION | 0.061578505 | 0.042052671 |
| GOCC_FICOLIN_1_RICH_GRANULE_LUMEN | 0.031653805 | 0.042225417 |
| GOCC_CONNEXIN_COMPLEX | 0.07021149 | 0.042532317 |
| GOMF_PHOSPHOLIPASE_INHIBITOR_ACTIVITY | 0.062274671 | 0.042801411 |
| GOBP_NEGATIVE_REGULATION_OF_HISTONE_METHYLATION | -0.032244558 | 0.043121136 |
| GOBP_HISTONE_H3_DEACETYLATION | -0.040915366 | 0.043791711 |
| GOMF_LRR_DOMAIN_BINDING | -0.030013304 | 0.043802393 |
| GOBP_REGULATION_OF_TRANSCRIPTION_INVOLVED_IN_G1_S_TRANSITION_OF_MITOTIC_CELL_CYCLE | 0.03214337 | 0.043975867 |
| GOBP_NEGATIVE_REGULATION_OF_RESPONSE_TO_OXIDATIVE_STRESS | 0.036987048 | 0.044096021 |
| GOBP_TYPE_B_PANCREATIC_CELL_DIFFERENTIATION | -0.048011222 | 0.044970321 |
| GOBP_SPLEEN_DEVELOPMENT | -0.031462457 | 0.045159279 |
| GOMF_VERY_LONG_CHAIN_FATTY_ACID_COA_LIGASE_ACTIVITY | 0.042449364 | 0.045692392 |
| GOBP_HISTONE_H3_K9_ACETYLATION | -0.028572407 | 0.045769647 |
| GOBP_RESPONSE_TO_PLATELET_DERIVED_GROWTH_FACTOR | 0.032748898 | 0.046153547 |
| GOBP_BLEB_ASSEMBLY | 0.110270902 | 0.046316748 |
| GOBP_NEGATIVE_REGULATION_OF_RESPONSE_TO_REACTIVE_OXYGEN_SPECIES | 0.035471591 | 0.046451451 |
| GOMF_TYPE_II_TRANSFORMING_GROWTH_FACTOR_BETA_RECEPTOR_BINDING | 0.058710032 | 0.046514646 |
| GOBP_BASE_CONVERSION_OR_SUBSTITUTION_EDITING | 0.035864673 | 0.046873944 |
| GOMF_GAP_JUNCTION_CHANNEL_ACTIVITY | 0.066759012 | 0.046883078 |
| GOBP_REGULATION_OF_CELL_GROWTH_INVOLVED_IN_CARDIAC_MUSCLE_CELL_DEVELOPMENT | 0.032648702 | 0.047402692 |
| GOCC_ENDOLYSOSOME_MEMBRANE | -0.02946887 | 0.047769062 |
| GOBP_REGULATION_OF_COMPLEMENT_DEPENDENT_CYTOTOXICITY | 0.068467857 | 0.047998045 |
| GOBP_REGULATION_OF_AMINO_ACID_TRANSPORT | 0.032528482 | 0.048073839 |
| GOBP_CARDIAC_MUSCLE_CELL_FATE_COMMITMENT | 0.095875943 | 0.048185295 |
| GOBP_REGULATION_OF_ERAD_PATHWAY | 0.028148593 | 0.049785668 |
